# SH2B1 Defends Against Energy Imbalance, Obesity, and Metabolic Disease via a Paraventricular Hypothalamus→Dorsal Raphe Nucleus Neurocircuit

**DOI:** 10.1002/advs.202400437

**Published:** 2024-06-17

**Authors:** Yuan Li, Min‐Hyun Kim, Lin Jiang, Lorelei Baron, Latrice D. Faulkner, David P. Olson, Xingyu Li, Noam Gannot, Peng Li, Liangyou Rui

**Affiliations:** ^1^ Department of Molecular & Integrative Physiology University of Michigan Medical School Ann Arbor MI 48109 USA; ^2^ College of Health Solutions Arizona State University Phoenix AZ 85004 USA; ^3^ Department of Pediatrics University of Michigan Medical School Ann Arbor MI 48109 USA; ^4^ Elizabeth Weiser Caswell Diabetes Institute University of Michigan Ann Arbor MI 48109 USA; ^5^ Life Sciences Institute University of Michigan Ann Arbor MI 48109 USA; ^6^ Department of Biologic and Materials Sciences School of Dentistry University of Michigan Ann Arbor MI 48109 USA; ^7^ Division of Gastroenterology and Hepatology Department of Internal Medicine University of Michigan Medical School Ann Arbor MI 48109 USA

**Keywords:** BDNF TrkB, dorsal raphe nucleus, energy balance, food intake, insulin leptin, obesity, paraventricular hypothalamus, SH2B1

## Abstract

SH2B1 mutations are associated with obesity, type 2 diabetes, and metabolic dysfunction‐associated steatotic liver disease (MASLD) in humans. Global deletion of Sh2b1 results in severe obesity, type 2 diabetes, and MASLD in mice. Neuron‐specific restoration of SH2B1 rescues the obesity phenotype of Sh2b1‐null mice, indicating that the brain is a main SH2B1 target. However, SH2B1 neurocircuits remain elusive. SH2B1‐expressing neurons in the paraventricular hypothalamus (PVH^SH2B1^) and a PVH^SH2B1^→dorsal raphe nucleus (DRN) neurocircuit are identified here. PVH^SH2B1^ axons monosynaptically innervate DRN neurons. Optogenetic stimulation of PVH^SH2B1^ axonal fibers in the DRN suppresses food intake. Chronic inhibition of PVH^SH2B1^ neurons causes obesity. In male and female mice, either embryonic‐onset or adult‐onset deletion of Sh2b1 in PVH neurons causes energy imbalance, obesity, insulin resistance, glucose intolerance, and MASLD. Ablation of Sh2b1 in the DRN‐projecting PVH^SH2B1^ subpopulation also causes energy imbalance, obesity, and metabolic disorders. Conversely, SH2B1 overexpression in either total or DRN‐projecting PVH^SH2B1^ neurons protects against diet‐induced obesity. SH2B1 binds to TrkB and enhances brain‐derived neurotrophic factor (BDNF) signaling. Ablation of Sh2b1 in PVH^SH2B1^ neurons induces BDNF resistance in the PVH, contributing to obesity. In conclusion, these results unveil a previously unrecognized PVH^SH2B1^→DRN neurocircuit through which SH2B1 defends against obesity by enhancing BDNF/TrkB signaling.

## Graphical abstract

SH2B1 deficiency induces obesity and metabolic disease in mice and humans. SH2B1‐expressing neurons in the PVH and a PVH^SH2B1^→DRN neurocircuit are uncovered here. Activation of the PVH^SH2B1^→DRN neurocircuit suppresses appetite. SH2B1 directly enhances BDNF signaling in PVH^SH2B1^ neurons, thereby increasing the ability of the PVH^SH2B1^→DRN neurocircuit to defend against energy imbalance, obesity, and metabolic disease.

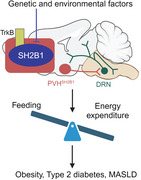

## 1 Introduction

Obesity‐associated genes predominantly regulate the central nervous system in humans, highlighting the pivotal role of the brain, particularly the hypothalamus, in obesity pathogenesis.^[^ ^1^ ^]^ The paraventricular hypothalamus (PVH) is long recognized as a satiety center, and ablation of PVH neurons causes hyperphagia and obesity.^[^ ^2^ ^]^ The PVH encompasses diverse neuronal subpopulations, which express distinct neuropeptides, receptors, and signaling proteins (e.g., oxytocin, prodynorphin, MC4R, GLP‐1R, and HTR2c).^[^ ^3^ ^]^ The PVH receives inputs from both intrahypothalamic and extrahypothalamic areas, and relays metabolic information to multiple downstream circuits.^[^ ^1^, ^4^ ^]^ In the acuate nucleus (ARC), POMC neurons and AgRP neurons, which sense circulating nutrient and hormonal cues, directly project to the PVH.^[^ ^5^ ^]^ In the PVH, MC4R neurons are a target of POMC and AgRP neurons to inhibit food intake.^[^ ^6^ ^]^ Several downstream targets of PVH neurons have been identified, including the parabrachial complex, central lateral parabrachial nucleus, and prelocus coeruleus.^[^ ^3^, ^7^ ^]^

SH2B1 is a SH2 domain‐containing protein and mediates cell signaling in response to various growth factors, hormones, and cytokines, including growth hormone, insulin, leptin, platelet‐derived growth factor, nerve growth factor (NGF), and brain‐derived neurotrophic factor (BDNF).^[^ ^8^ ^]^ In mice, global deletion of Sh2b1 causes hyperphagia, obesity, type 2 diabetes, and metabolic dysfunction‐associated steatotic liver disease (MASLD, previously called NAFLD).^[^ ^9^ ^]^ Likewise, human SH2B1 mutations are also linked to obesity, type 2 diabetes, and MASLD.^[^ ^8^, ^10^ ^]^ Importantly, neuron‐specific restoration of SH2B1 completely rescues the hyperphagic and obese phenotypes of Sh2b1‐null mice.^[^ ^11^ ^]^ These observations suggest that neuronal SH2B1 is required for defense against energy imbalance and obesity in both humans and rodents. To explore SH2B1 pathways in vivo, we deleted Sh2b1 specifically in leptin receptor (LepRb) expressing neurons. LepRb neuron‐specific ablation of Sh2b1 abrogates the ability of leptin to stimulate sympathetic nerve activity, suppresses brown adipose tissue (BAT) thermogenesis, and causes obesity, insulin resistance, and MASLD.^[^ ^12^ ^]^ Unexpectedly, food intake is normal in the mutant mice; hence, SH2B1 neurons, which regulate appetite, remain unknown. In cell culture, SH2B1 binds to TrkB to enhance BDNF signaling.^[^ ^8^, ^13^ ^]^ *BDNF* or *TrkB* mutations phenocopy SH2B1 mutations in patients.^[^ ^1^, ^14^ ^]^ These observations raise the possibility that SH2B1 may regulate appetite and food intake by enhancing the BDNF/TrkB pathway in hypothalamic neurocircuits.

The dorsal raphe nucleus (DRN) emerges as an important neural center to control energy balance, body weight, and metabolism.^[^ ^15^ ^]^ DRN neurons receive inputs from both the forebrain and the hindbrain and project to multiple downstream brain areas.^[^ ^3^, ^15^, ^16^ ^]^ The DRN encompasses serotonergic (DRN^5‐HT^), glutamatergic (DRN^Vglut3^), and GABAergic (DRN^Vgat^) neurons. The DRN^5‐HT^ and DRN^Vglut3^ circuits are anorexigenic, whereas the DRN^Vgat^ pathway is orexigenic.^[^ ^15^ ^]^ DRN^5‐HT^ neurons directly activate POMC neurons via 5‐HT_2C_R in the ARC to reduce food intake.^[^ ^16^, ^17^ ^]^ Stimulation of the DRN^5‐HT^→ARC circuit inhibits feeding in mice.^[^ ^16j^ ^]^ DRN^5‐HT^ neurons also project to the ventral tegmental area (VTA), lateral hypothalamus (LH), and bed nucleus of the stria terminalis (BNST).^[^ ^16j,k^ ^]^ In addition, DRN^5‐HT^ neurons promote adipose thermogenesis and defend against hyperglycemia and hyperlipidemia.^[^ ^18^ ^]^ Like DRN^5‐HT^ neurons, DRN^Vglut3^ neurons also suppress appetite and food intake.^[^ ^16d,e^ ^]^ By contrast, DRN^Vgat^ neurons promote feeding.^[^ ^16e,n^ ^]^ Additionally, DRN^Vgat^ neurons suppress BAT thermogenesis and energy expenditure.^[^ ^16b^ ^]^ In this study, we show that SH2B1‐expressing neurons in the PVH (PVH^SH2B1^) project to the DRN. Optogenetic activation of the PVH^SH2B1^→DRN circuit suppresses appetite. Deletion of Sh2b1 in DRN‐projecting PVH^SH2B1^ neurons causes obesity, insulin resistance, and MASLD. SH2B1 counteracts energy imbalance and obesity at least in part by enhancing BDNF action. These results unveil a previously unrecognized PVH^SH2B1^→DRN neurocircuit as well as SH2B1‐based mechanism shaping circuit activity.

## 2 Results

### 2.1 Deletion of Sh2b1 in PVH Neurons Causes Obesity

**Figure 1:**
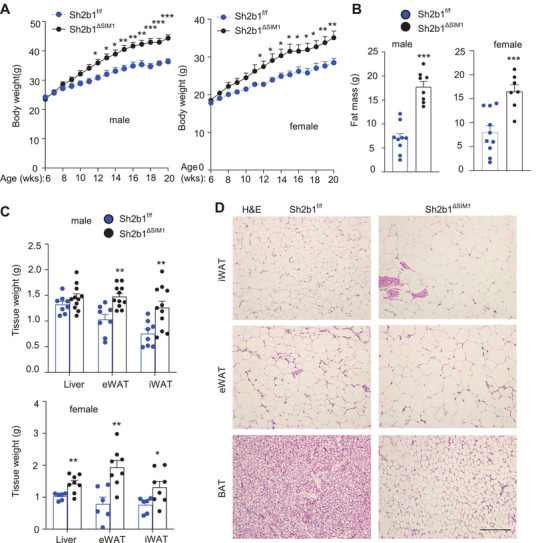
SIM1 neuron‐specific deletion of Sh2b1 results in obesity. A) Growth curves. Male Sh2b1*^f/f^*: *n* = 9, male Sh2b1*^ΔSIM1^*: *n* = 15, female Sh2b1*^f/f^*: *n* = 8, female Sh2b1*^ΔSIM1^*: *n* = 9. B) Total fat mass at 18 weeks of age. Male Sh2b1*^f/f^*: *n* = 10, male Sh2b1*^ΔSIM1^*: *n* = 8, female Sh2b1*^f/f^*: *n* = 9, female Sh2b1*^ΔSIM1^*: *n* = 7. C) Tissue weight at 20 weeks of age. Male Sh2b1*^f/f^*: *n* = 8, male Sh2b1*^ΔSIM1^*: *n* = 11, female Sh2b1*^f/f^*: *n* = 6, female Sh2b1*^ΔSIM1^*: *n* = 8. D) H&E staining (three males per group at 20 weeks of age). Scale bar: 200 µm. Data are presented as mean ± SEM. *p < 0.05, **p < 0.01, ***p < 0.001, B, C) 2‐tailed unpaired Student's *t‐*test or A) two‐way ANOVA.

To explore the SH2B1 function in the PVH, we generated SIM1 neuron‐specific Sh2b1 knockout mice (Sh2b1*^ΔSIM1^*) by crossing Sh2b1*^f/f^* mice with *Sim1‐Cre* mice. Sh2b1*^f/f^* and *Sim1‐Cre* mice were described previously.^[^ ^6^, ^19^ ^]^ To validate PVH‐targeting, *Sim1‐Cre* mice were crossed with Cre‐dependent green fluorescent protein (GFP) reporter mice. GFP expression was restricted to the PVH (Figure S1A, Supporting Information). To further confirm PVH‐specific deletion of Sh2b1, hypothalamic sections were stained with anti‐SH2B1 antibody. SH2B1 immunoreactivity was substantially reduced in the PVH but not in other hypothalamic regions of Sh2b1*^ΔSIM1^* mice, compared to Sh2b1*^f/f^* mice (Figure S1B, Supporting Information). We monitored the body weights of these mice on a normal chow diet. Remarkably, Sh2b1*^ΔSIM1^* males and females developed severe obesity (**Figure** **1**A). Whole body fat mass was significantly higher in Sh2b1*^ΔSIM1^* mice relative to sex‐matched Sh2b1*^f/f^* littermates (Figure 1B). Epididymal white adipose tissue (eWAT) and inguinal WAT (iWAT) weights were markedly higher in Sh2b1*^ΔSIM1^* than in Sh2b1*^f/f^* males (Figure 1C). Adipocyte size in iWAT and eWAT was substantially larger in Sh2b1*^ΔSIM1^* than in Sh2b1*^f/f^* littermates (Figure 1D). In Sh2b1*^ΔSIM1^* female mice, gonadal WAT (gWAT) and iWAT weights were also significantly higher and lipid droplets were dramatically larger (Figure 1C; Figure S1C, Supporting Information). Lipid droplets in BAT were larger in Sh2b1*^ΔSIM1^* mice (Figure 1D; Figure S1C, Supporting Information). These results demonstrate that SIM1 neuron‐intrinsic SH2B1 is required for counteracting obesity.

### 2.2 Sh2b1*^ΔSIM1^* Mice Develop Glucose Intolerance, Insulin Resistance, and MASLD

**Figure 2:**
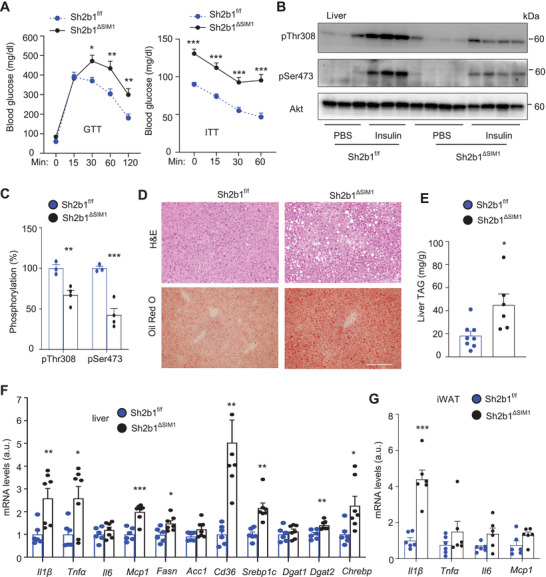
Sh2b1*^ΔSIM1^* mice develop glucose intolerance, insulin resistance, and MASLD. A) GTT (Sh2b1*^f/f^*: *n* = 8, Sh2b1*^ΔSIM1^*: *n* = 11) and ITT (Sh2b1*^f/f^*: *n* = 8, Sh2b1*^ΔSIM1^*: *n* = 8) at 15 weeks of age. B,C) Mice (20 weeks old) were fasted overnight and injected with insulin. Liver extracts were immunoblotted with the indicated antibodies. AKT phosphorylation was normalized to total AKT levels. Sh2b1*^f/f^*: *n* = 3, Sh2b1*^ΔSIM1^*: *n* = 4. D) H&E and Oil Red O staining of liver sections at 18 weeks of age (three mice per group). Scale bar: 200 µm. E) Liver TAG levels at 20 weeks of age (normalized to liver weight). Sh2b1*^f/f^*: *n* = 8, Sh2b1*^ΔSIM1^*: *n* = 6. F,G) mRNA abundance at 20 weeks of age (normalized to 36B4 levels). Liver Sh2b1*^f/f^*: *n* = 6, liver Sh2b1*^ΔSIM1^*: *n* = 7. iWAT Sh2b1*^f/f^*: *n* = 6, iWAT Sh2b1*^ΔSIM1^*: *n* = 6. a.u.: arbitrary units. All data were from male mice. Data are presented as mean ± SEM. *p < 0.05, **p < 0.01, ***p < 0.001, C,E–G) 2‐tailed unpaired Student's *t‐*test or A) two‐way ANOVA (A).

We next examined the metabolic function of PVH‐intrinsic SH2B1 and performed glucose tolerance test (GTT) and insulin tolerance test (ITT) on Sh2b1*^ΔSIM1^* and Sh2b1*^f/f^* mice at 15 weeks of age (on a chow diet). Blood glucose levels were significantly higher in Sh2b1*^ΔSIM1^* males and females relative to sex‐matched Sh2b1*^f/f^* mice (**Figure** **2**A; Figure S2A, Supporting Information). Mice were fasted overnight and injected with insulin. Insulin‐stimulated phosphorylation of hepatic AKT (pThr308, pSer473) was markedly lower in Sh2b1*^ΔSIM1^* than in Sh2b1*^f/f^* mice (Figure 2B,C). Hepatic lipid droplets were larger and more abundant and liver triacylglycerol (TAG) levels were significantly higher in Sh2b1*^ΔSIM1^* males and females relative to sex‐matched Sh2b1*^f/f^* mice (Figure 2D,E; Figure S2B, Supporting Information). Liver expression of lipogenic genes (*Srebp1c, Chrebp, Fasn*) and lipid uptake gene (*CD36*) was higher in Sh2b1*^ΔSIM1^* mice (Figure 2F). Considering that chronic inflammation promotes metabolic disorders, we accessed cytokine expression. Liver expression of *Tnfα*, *Il1β*, and *Mcp1* and adipose expression of *Il1β* were significantly higher in Sh2b1*^ΔSIM1^* than in Sh2b1*^f/f^* mice (Figure 2F,G). Of note, GTT and ITT were comparable between Sh2b1*^ΔSIM1^* and Sh2b1*^f/f^* mice prior to their body weight divergence (Figure S2C,D, Supporting Information). These data demonstrate that disruption of Sh2b1 in SIM1 neurons results in obesity‐associated insulin resistance, glucose intolerance, and MASLD.

### 2.3 Deletion of Sh2b1 in PVH Neurons Induces Energy Imbalance

**Figure 3:**
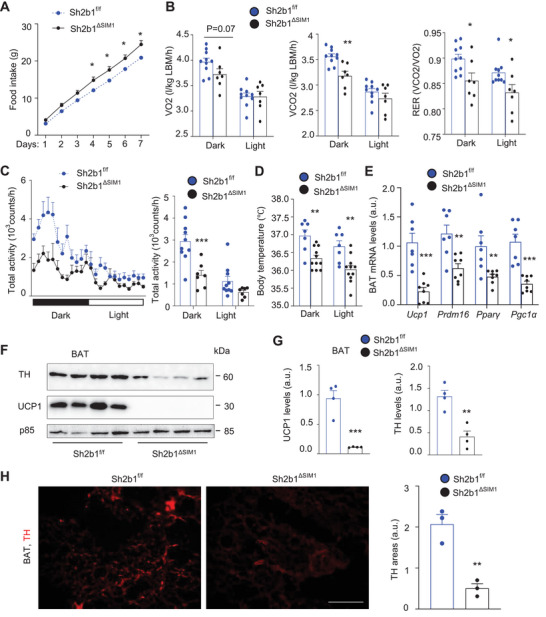
SIM1 neuron‐specific deletion of Sh2b1 disrupts energy balance. A) Food intake at 9 weeks of age. Sh2b1*^f/f^*: *n* = 6, Sh2b1*^ΔSIM1^*: *n* = 6. B,C) O_2_ consumption, CO_2_ production (normalized to lean mass), RER, and locomotor activity at 10 weeks of age. Sh2b1*^f/f^*: *n* = 10, Sh2b1*^ΔSIM1^*: *n* = 7. D) Body core temperatures at 9 weeks of age. Sh2b1*^f/f^*: *n* = 7, Sh2b1*^ΔSIM1^*: *n* = 11. E) mRNA abundance at 20 weeks of age (normalized to 36B4 levels). Sh2b1*^f/f^*: *n* = 7, Sh2b1*^ΔSIM1^*: *n* = 8. a.u.: arbitrary units. F,G) BAT extracts were immunoblotted with the indicated antibodies (20 weeks old). UCP1 and TH levels were normalized to p85 levels four mice per group). H) BAT sections were stained with anti‐TH antibody (three mice per group at 20 weeks of age). Scale bar: 200 µm. All data were from male mice. Data are presented as mean ± SEM. *p < 0.05, **p < 0.01, ***p < 0.001, B, D, E, G) 2‐tailed unpaired Student's *t‐*test, or A, C) two‐way ANOVA.

We sought to interrogate the underlying mechanism of obesity. We examined energy balance by CLAMS in Sh2b1*^f/f^* and Sh2b1*^ΔSIM1^* littermates (on a chow diet) at 9 weeks of age when their body weights began to diverge (Figure S3A, Supporting Information). Food intake was significantly higher in Sh2b1*^ΔSIM1^* than in Sh2b1*^f/f^* littermates (**Figure** **3**A). In the dark phase, O_2_ consumption and CO_2_ production (normalized to lean mass) were considerably lower in Sh2b1*^ΔSIM1^* than in Sh2b1*^f/f^* mice (Figure 3B; Figure S3B, Supporting Information). The respiratory exchange ratio (RER) was significantly lower in Sh2b1*^ΔSIM1^* than in Sh2b1*^f/f^* mice (Figure 3B; Figure S3B, Supporting Information). Analysis of covariance (ANCOVA) further confirmed a decrease in energy expenditure in Sh2b1*^ΔSIM1^* mice (Figure S3C, Supporting Information). Locomotor activity (dark phase) and body core temperature (both dark and light phases) were significantly lower in Sh2b1*^ΔSIM1^* than in Sh2b1*^f/f^* mice (Figure 3C,D). BAT expression of thermogenic genes (*Ucp1, Prdm16, Pparγ, Pgc1α*) was markedly lower in Sh2b1*^ΔSIM1^* than in Sh2b1*^f/f^* mice (Figure 3E). UCP1 protein levels were dramatically lower in Sh2b1*^ΔSIM1^* mice (Figure 3F,G). BAT sections were stained with antibodies to tyrosine hydroxylase (TH), a sympathetic nerve marker. TH^+^ fibers and puncta (cross‐sections) were dramatically reduced in Sh2b1*^ΔSIM1^* mice compared to Sh2b1*^f/f^* littermates (Figure 3H). TH protein levels in BAT extracts were significantly lower in Sh2b1*^ΔSIM1^* mice (Figure 3F,G). These results suggest that PVH^SH2B1^ neuron‐intrinsic SH2B1 suppresses food intake while increasing energy expenditure.

### 2.4 Adult‐Onset Deletion of Sh2b1 in PVH Neurons Causes Obesity and Metabolic Disorders

**Figure 4:**
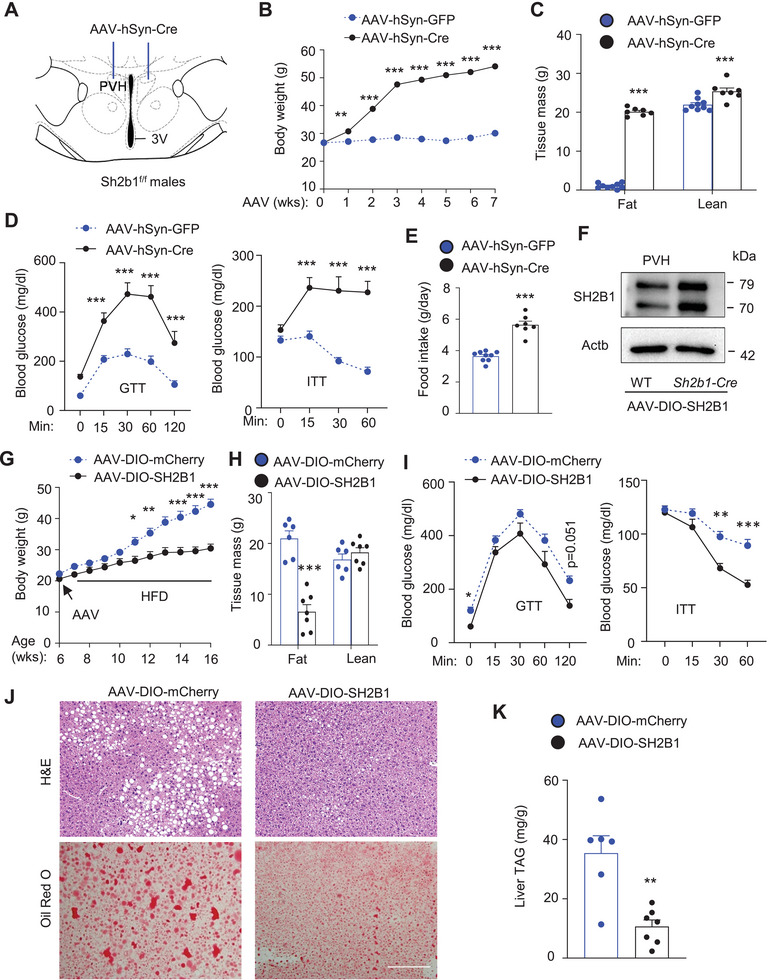
Effects of adult‐onset ablation or overexpression of SH2B1 in PVH neurons on body weight and metabolism. A–E) Sh2b1*^f/f^* males were bilaterally microinjected into the PVH with AAV1‐hSyn‐Cre (*n* = 7) or AAV1‐hSyn‐GFP (*n* = 9) and fed a normal chow diet. B) Growth curves. C) Total fat and lean mass 6 weeks post AAV transduction. D) GTT and ITT 7 weeks after AAV transduction. E) Food intake 10 weeks after AAV transduction. F) WT and Sh2b1*‐Cre* mice were microinjected into the PVH with AAV9‐CAG‐DIO‐SH2B1 vector. PVH extracts were immunoblotted with the indicated antibodies (repeated in two pairs). G–K) Sh2b1*‐Cre* males were bilaterally microinjected into the PVH with AAV9‐CAG‐DIO‐SH2B1 or AAV9‐CAG‐DIO‐mCherry vector. One week later, mice were fed an HFD. G) Growth curves. AAV‐DIO‐mCherry: *n* = 6, AAV‐DIO‐SH2B1: *n* = 7. H) Total fat and lean mass 9 weeks post AAV injection. AAV‐DIO‐mCherry: *n* = 6, AAV‐DIO‐SH2B1: *n* = 7. I) GTT and ITT in 10 weeks post AAV transduction. AAV‐DIO‐mCherry: *n* = 6, AAV‐DIO‐SH2B1: *n* = 6. J) H&E and Oil red O staining of liver sections at 10 weeks of age. Scale bar: 200 µm. K) Liver TAG levels (normalized to liver weight) 10 weeks post AAV transduction. AAV‐DIO‐mCherry: *n* = 6, AAV‐DIO‐SH2B1: *n* = 7. Data are presented as mean ± SEM. *p < 0.05, **p < 0.01, ***p < 0.001, C, E, H, K) 2‐tailed unpaired Student's *t‐*test or B, D, G, I) two‐way ANOVA.

Cre is also expressed in a few non‐PVH areas in *Sim1‐Cre* mice,^[^ ^6^ ^]^ raising a concern that in Sh2b1*^ΔSIM1^* mice, Sh2b1 might be also deleted in these non‐PVH regions. To address this concern, adult Sh2b1*^f/f^* males (8 weeks old) were bilaterally microinjected into the PVH with adeno‐associated virus (AAV1)‐hSyn‐Cre (deleting Sh2b1 in PVH neurons) or AAV1‐hSyn‐GFP vector (control) (**Figure** **4**A; Figure S4A, Supporting Information). Body weight diverged between the two groups within one week after AAV injection (Figure 4B). AAV1‐hSyn‐Cre mice gained over 27 g of body weight within 7 weeks, whereas AAV1‐hSyn‐GFP mice gained ≈3.3 g. Total fat mass, but not lean mass, was drastically higher in AAV1‐hSyn‐Cre than in AAV1‐hSyn‐GFP mice 5 weeks after AAV injection (Figure 4C). In GTT and ITT, blood glucose levels were markedly higher in AAV1‐hSyn‐Cre than in AAV‐hSyn‐GFP mice 6 weeks after AAV transduction (Figure 4D). Food intake was significantly higher in AAV1‐hSyn‐Cre mice (Figure 4E). AAV1‐hSyn‐Cre mice displayed cold intolerance (Figure S4B, Supporting Information). These results confirm that PVH neuron‐specific deletion of Sh2b1 causes hyperphagia, obesity, and metabolic disorders independently of brain development.

### 2.5 PVH^SH2B1^ Neuron‐Specific Overexpression of SH2B1 Protects Against Diet‐Induced Obesity

To determine whether overexpression of SH2B1 confers metabolic benefits, we prepared an AAV‐CAG‐DIO‐SH2B1 vector expressing human SH2B1β in a Cre‐dependent manner (Figure S4C, Supporting Information). AAV‐CAG‐DIO‐SH2B1 plasmids were cotransfected with or without Cre plasmids into N2a cells, a neuronal line. SH2B1 was detected in the presence, but not in the absence, of Cre (Figure S4D, Supporting Information). To validate the viral vector in vivo, the AAV9‐CAG‐DIO‐SH2B1 vector was co‐microinjected into mouse cortices with or without the AAV‐hSyn‐Cre vector. Cre expression substantially increased cortical SH2B1 levels (Figure S4E, Supporting Information). To deliver SH2B1 specifically into PVH^SH2B1^ neurons, Sh2b1*‐IRES‐eGFP‐2A‐Cre* (referred to as Sh2b1*‐Cre*) mice were bilaterally microinjected into the PVH with AAV9‐CAG‐DIO‐SH2B1 vector. Wild‐type (WT) mice were used as controls. In Sh2b1*‐Cre* mice, an *IRES‐eGFP‐2A‐Cre* gene cassette was knocked in the Sh2b1 locus (after the STOP codon) to generate GFP and Cre under the control of the endogenous Sh2b1 promoter.^[^ ^12^ ^]^ Indeed, endogenous SH2B1 and GFP were colocalized in PVH neurons (Figure S4F, Supporting Information). AAV9‐CAG‐DIO‐SH2B1 transduction increased SH2B1 levels in the PVH in Sh2b1*‐Cre* mice relative to WT mice (Figure 4F). To visually confirm PVH^SH2B1^ neuron‐specific targeting, Sh2b1*‐Cre* mice were bilaterally injected into the PVH with a Cre‐dependent AAV9‐CAG‐DIO‐mCherry reporter. PVH^SH2B1^ neurons were marked by mCherry (Figure S4G, Supporting Information).

Sh2b1*‐Cre* males were bilaterally microinjected into the PVH with AAV9‐CAG‐DIO‐SH2B1 (PVH^SH2B1^ neuron‐specific overexpression of SH2B1) or AAV9‐CAG‐DIO‐mCherry (control). One week later, mice were fed a high‐fat diet (HFD). Body weight diverged between the two groups 3 weeks after AAV injection and became dramatically lower in AAV9‐CAG‐DIO‐SH2B1 mice relative to AAV9‐CAG‐DIO‐mCherry mice (Figure 4G). Total fat mass and iWAT weight, but not lean mass, was significantly lower in AAV9‐CAG‐DIO‐SH2B1 mice 6 weeks after AAV transduction (Figure 4H; Figure S4G, Supporting Information). Adipocyte size in eWAT and iWAT was smaller in AAV9‐CAG‐DIO‐SH2B1 mice (Figure S4I, Supporting Information). Glucose intolerance and insulin resistance were improved in AAV9‐CAG‐DIO‐SH2B1 mice as assessed by GTT and ITT, respectively (Figure 4I). Liver weight and liver TAG were significantly lower, and hepatic lipid droplets were substantially smaller and less abundant in AAV9‐CAG‐DIO‐SH2B1 mice relative to AAV9‐CAG‐DIO‐mCherry (Figure 4J,K; Figure S4H, Supporting Information). These findings point to a new anti‐obesity strategy by raising SH2B1 levels in PVH^SH2B1^ neurons.

### 2.6 PVH^SH2B1^ Neurons Project to the DRN

**Figure 5:**
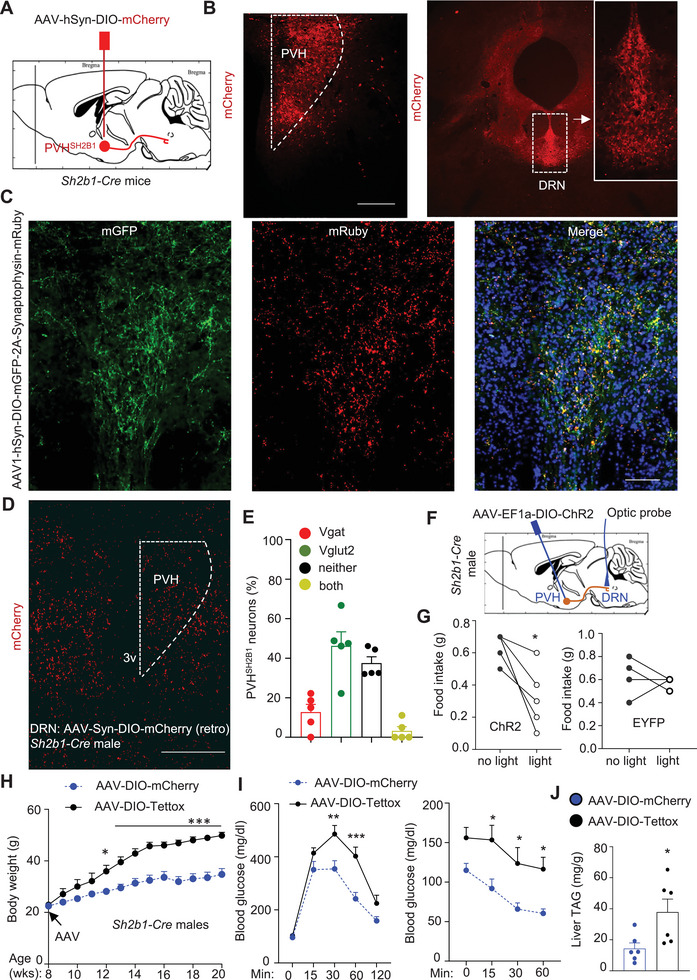
PVH^SH2B1^→DRN neurocircuit inhibits food intake and obesity. A,B) Sh2b1*‐Cre* males were unilaterally microinjected into the PVH with AAV9‐hSyn‐DIO‐mCherry vector. Brain mCherry fluorescence was assessed 4 weeks post AAV injection. Scale bar: 500 µm. C) Sh2b1*‐Cre* mice were microinjected into the PVH with AAV1‐hSyn‐DIO‐mGFP‐2A‐Synaptophysin‐mRuby vector, and GFP and mRuby were visualized 4 weeks later. Scale bar: 100 µm. D,E) Sh2b1*‐Cre* males were microinjected into the DRN with retrograde AAV‐hSyn‐DIO‐mCherry vector at 9 weeks of age. Hypothalamic mCherry, Vglut2, and Vgat were assessed by RNAscope 5 weeks later. D) *mCherry* ^+^ neurons. Scale bar: 500 µm. E) mCherry^+^ subpopulations in the PVH (normalized to total mCherry^+^ neurons, *n* = 5 mice). F,G) Sh2b1*‐Cre* males were microinjected into the PVH with AAV5‐hSyn‐DIO‐ChR2 (*n* = 6) or AAV5‐hSyn‐DIO‐GFP vector (*n* = 6). An optic probe was placed in the DRN and delivered blue light for 15 min. Food intake was measured before and after optic stimulation. H–J) Sh2b1*‐Cre* males were bilaterally microinjected into the PVH with AAV9‐hSyn‐DIO‐Tettox or AAV9‐hSyn‐DIO‐mCherry vector and fed an HFD. H) Growth curves. AAV‐hSyn‐DIO‐mCherry: *n* = 8, AAV‐hSyn‐DIO‐Tettox: *n* = 8. I) GTT and ITT 6 weeks after AAV transduction. AAV‐hSyn‐DIO‐mCherry: *n* = 6 for GTT and ITT, AAV‐hSyn‐DIO‐Tettox: *n* = 8 for GTT and *n* = 7 for ITT. J) Liver TAG levels (normalized to liver weight) 12 weeks post AAV transduction. AAV‐hSyn‐DIO‐mCherry: *n* = 6, AAV‐hSyn‐DIO‐Tettox: *n* = 6. Data are presented as mean ± SEM. *p < 0.05, **p < 0.01, ***p < 0.001, G) paired Student's *t‐*test, J) 2‐tailed unpaired Student's *t‐*test, H, I) or two‐way ANOVA.

We set out to map PVH^SH2B1^ neurocircuits. Sh2b1*‐Cre* males were unilaterally microinjected into the PVH with an AAV9‐hSyn‐DIO‐mCherry vector (**Figure** **5**A). PVH^SH2B1^ neuronal somas were marked by mCherry as expected (Figure 5B). PVH^SH2B1^ axonal fibers (mCherry^+^) were very abundant in the DRN (Figure 5B). PVH^SH2B1^ projections to the periaqueductal gray (PAG) were also detected but to a substantially lower level. To examine monosynaptic connectivity, Sh2b1*‐Cre* males were microinjected into the PVH with a Cre‐dependent AAV1‐hSyn‐DIO‐mGFP‐2A‐Synaptophysin‐mRuby vector. Synaptophysin is an integral membrane protein of presynaptic vesicles, and synaptophysin‐mRuby protein localizes in nerve terminals to mark synapse formation.^[^ ^20^ ^]^ We detected abundant synaptophysin‐mRuby puncta in the DRN (Figure 5C). These results suggest that PVH^SH2B1^ neurons form monosynaptic connections with DRN neurons.

To analyze PVH^SH2B1^ neuron phenotypes, Sh2b1*‐Cre* mice were microinjected into the DRN with retrograde, *Cre*‐dependent AAV‐hSyn‐DIO‐mCherry vector to mark DRN‐projecting PVH^SH2B1^ neurons by mCherry (Figure S5A, Supporting Information). Vglut2^+^ glutamatergic neurons, Vgat^+^ GABAergic neurons, and mCherry^+^ neurons were identified by RNAscope. We detected mCherry^+^ neurons in the PVH (Figure 5D), confirming the PVH^SH2B1^→DRN neurocircuit. Glutamatergic PVH^SH2B1^ neurons (Vglut2^+^) were ≈3.6‐fold higher relative to GABAergic PVH^SH2B1^ neurons (Vgat^+^) (Figure 5E; Figure S5B, Supporting Information). These data are in line with the previous reports that PVH neurons are predominantly glutamatergic.^[^ ^7^, ^21^ ^]^ We also detected Vglut2^−^Vgat^−^ double‐negative and Vglut2^+^Vgat^+^ double‐positive PVH^SH2B1^ neurons (Figure 5E).

We next examined PVH^SH2B1^ neuronal activation by measuring the expression of c‐Fos, a commonly used neuronal activation marker. In wild‐type mice, c‐Fos^+^ neurons in the PVH were higher in the fed than in the fasted conditions (Figure S5C, Supporting Information). Importantly, PVH c‐Fos^+^ neurons were significantly lower in Sh2b1*^ΔSIM1^* than in Sh2b1*^f/f^* mice under fed conditions (Figure S5D,E, Supporting Information). These results raise the possibility that PVH^SH2B1^ neuron‐intrinsic SH2B1 is involved in central metabolic sensing and integration.

### 2.7 The PVH^SH2B1^→DRN Neurocircuit Safeguards Appetite and Body Weight

To determine whether the PVH^SH2B1^→DRN circuit regulates appetite, Sh2b1*‐Cre* males were bilaterally microinjected into the PVH with Cre‐dependent AAV5‐hSyn‐DIO‐ChR2 or AAV5‐hSyn‐DIO‐GFP (control) vector. An optic fiber was implanted in the DRN (Figure 5F). Four weeks later, mice were fasted overnight. PVH^SH2B1^ axonal fibers in the DRN were then stimulated with blue light. Optogenetic stimulation significantly suppressed food intake in AAV5‐hSyn‐DIO‐ChR2 but not AAV5‐hSyn‐DIO‐GFP mice (Figure 5G). To test if chronic inhibition of PVH^SH2B1^ neurons causes obesity, Sh2b1*‐Cre* mice were bilaterally microinjected into the PVH with Cre‐dependent AAV9‐hSyn‐DIO‐Tetanus toxin (Tettox) or AAV9‐hSyn‐DIO‐mCherry (control) vector (Figure S6A, Supporting Information). Tettox silences synaptic transmissions.^[^ ^22^ ^]^ Body weight diverged after 2 weeks of AAV transduction and then, became progressively and dramatically higher in AAV‐hSyn‐DIO‐Tettox mice relative to AAV9‐hSyn‐DIO‐mCherry mice (Figure 5H). Fat content, iWAT weight, and liver weight were significantly higher, and body core temperature (dark phase) was lower in AAV9‐hSyn‐DIO‐Tettox than in AAV9‐hSyn‐DIO‐mCherry mice (Figure S6A–C, Supporting Information). AAV9‐hSyn‐DIO‐Tettox mice developed glucose intolerance and insulin resistance, as assessed by GTT and ITT, compared to AAV9‐hSyn‐DIO‐mCherry mice (Figure 5I). Liver TAG levels were higher, and liver lipid droplets were larger and more abundant in AAV9‐hSyn‐DIO‐Tettox mice (Figure 5J; Figure S6D, Supporting Information). These results suggest that PVH^SH2B1^ neurons deliver stimulatory inputs to the DRN to counteract hyperphagia and obesity.

### 2.8 Deletion of Sh2b1 in DRN‐Projecting PVH^SH2B1^ Neurons Causes Obesity and Metabolic Disorders

**Figure 6:**
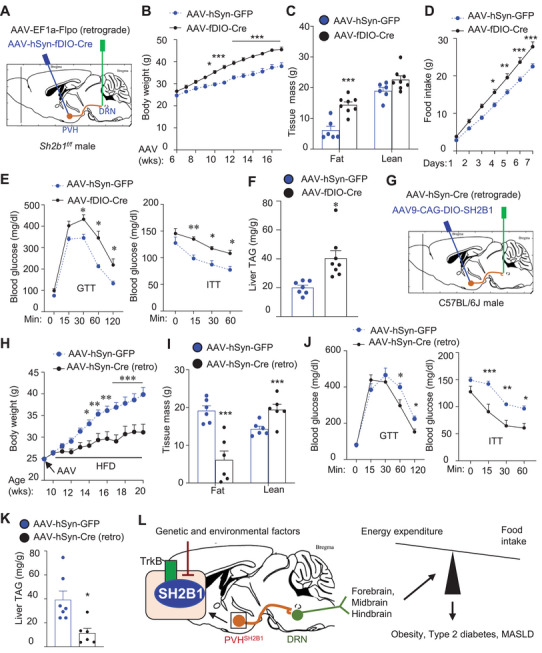
SH2B1 in DRN‐projecting PVH^SH2B1^ neurons defends against obesity and metabolic disorders. A–F) Sh2b1*^f/f^* males were microinjected into the PVH with AAV8‐hSyn‐fDIO‐Cre or AAV‐EF1a‐GFP bilaterally while retrograde AAV‐EF1a‐Flpo vector was injected into the DRN. Mice were on a normal chow diet. B) Growth curves. AAV‐EF1a‐GFP: *n* = 7, AAV‐hSyn‐fDIO‐Cre: *n* = 10. C) Total fat mass 6 weeks after AAV transduction. AAV‐EF1a‐GFP: *n* = 6, AAV‐hSyn‐fDIO‐Cre: *n* = 8. D) Food intake 7 weeks post AAV transduction. AAV‐EF1a‐GFP: *n* = 6, AAV‐hSyn‐fDIO‐Cre: *n* = 7. E) GTT and ITT 8 weeks after AAV transduction. AAV‐EF1a‐GFP: *n* = 7, AAV‐hSyn‐fDIO‐Cre: *n* = 6. F) Liver TAG levels (normalized to liver weight) 10 weeks post AAV injection. AAV‐EF1a‐GFP: *n* = 7, AAV‐hSyn‐fDIO‐Cre: *n* = 8. G–K) C57BL/6J males were bilaterally microinjected into the PVH with AAV9‐CAG‐DIO‐SH2B1 vector while retrograde AAV‐hSyn‐Cre vector was injected into the DRN. Mice were fed an HFD. H) Growth curves. AAV‐hSyn‐GFP: *n* = 7, AAV‐hSyn‐Cre: *n* = 8. I) Total fat mass 7 weeks after AAV transduction. AAV‐hSyn‐GFP: *n* = 6, AAV‐hSyn‐Cre: *n* = 6. J) GTT and ITT 8 weeks after AAV transduction. AAV‐hSyn‐GFP: *n* = 6, AAV‐hSyn‐Cre: *n* = 7 for GTT and *n* = 6 for ITT. K) Liver TAG levels (normalized to liver weight) 11 weeks post AAV injection. AAV‐hSyn‐GFP: *n* = 7, AAV‐hSyn‐Cre: *n* = 6. L) Genetic and/or environmental factors inhibit the ability of SH2B1 to enhance BDNF/TrkB pathway in PVH^SH2B1^ neurons, thereby impairing the PVH^SH2B1^→DRN neurocircuit. DRN neurons project to the hindbrain, midbrain, and forebrain to defend energy balance and body weight. Impairment in the PVH^SH2B1^→DRN neurocircuit promotes energy imbalance, obesity, type 2 diabetes, and MASLD. Data are presented as mean ± SEM. *p < 0.05 **p < 0.01, ***p < 0.001, C, F, I, K) 2‐tailed unpaired Student's *t‐*test or B, D, E, H, J) two‐way ANOVA.

To delete Sh2b1 specifically in the DRN‐projecting PVH^SH2B1^ subpopulation, we generated a Flpo‐dependent AAV‐hSyn‐fDIO‐Cre vector in which an inverse *Cre* gene was flanked by double Frt sites (Figure S7A, Supporting Information). Flpo flippase induces *Cre* inversion to trigger Cre expression. Sh2b1*^f/f^* males (on chow diet) were bilaterally microinjected into the PVH with AAV8‐hSyn‐fDIO‐Cre or AAV8‐hSyn‐GFP (control) vector while the DRN was injected with retrograde AAV‐EF1a‐Flpo vector (**Figure** **6**A). AAV‐EF1a‐Flpo virus transduced PVH^SH2B1^ axonal terminals in the DRN and was retrogradely transferred to PVH^SH2B1^ somas (transduced with AAV8‐hSyn‐fDIO‐Cre vector) to trigger Cre expression. Cre in turn induced deletion of Sh2b1 specifically in DRN‐projecting PVH^SH2B1^ neurons. Body weight diverged within 3 weeks of AAV transduction and then became progressively higher in AAV8‐hSyn‐fDIO‐Cre mice relative to AAV8‐hSyn‐GFP mice (Figure 6B). Total fat mass was significantly higher in AAV8‐hSyn‐fDIO‐Cre mice relative to AAV8‐hSyn‐GFP mice 6 weeks after AAV transduction (Figure 6C). White adipocyte size was larger in AAV8‐hSyn‐fDIO‐Cre mice (Figure S7B, Supporting Information). Food intake was significantly higher in AAV8‐hSyn‐fDIO‐Cre than in AAV8‐hSyn‐GFP mice (Figure 6D). Body core temperature (dark phase) was significantly lower in AAV8‐hSyn‐fDIO‐Cre mice (Figure S7C, Supporting Information). Deletion of Sh2b1 specifically in DRN‐projecting PVH^SH2B1^ neurons also resulted in glucose intolerance and insulin resistance, as assessed in GTT and ITT (Figure 6E). Liver TAG levels were significantly higher in AAV8‐hSyn‐fDIO‐Cre than in AAV‐hSyn‐GFP mice (Figure 6F). Liver lipid droplets were larger and more abundant in AAV8‐hSyn‐fDIO‐Cre mice (Figure S7B, Supporting Information).

### 2.9 Overexpression of SH2B1 in DRN‐Projecting PVH Neurons Protects Against HFD‐Induced Obesity

C57BL/6J males were bilaterally microinjected into the PVH with Cre‐dependent AAV9‐CAG‐DIO‐SH2B1 vector while the DRN was injected with retrograde AAV‐hSyn‐Cre or AAV‐hSyn‐GFP (control) vector (Figure 6G). This paradigm resulted in SH2B1 overexpression specifically in a PVH subpopulation projecting to the DRN. Mice were fed an HFD after one week of recovery. Body weight diverged in 2 weeks after AAV transduction and then became progressively and markedly lower in AAV‐hSyn‐Cre mice (SH2B1 overexpression) relative to AAV‐hSyn‐GFP mice (Figure 6H). Total fat mass was significantly lower in AAV‐hSyn‐Cre mice relative to AAV‐hSyn‐GFP mice 7 weeks after HFD (Figure 6I). AAV‐hSyn‐Cre mice displayed significant improvements in glucose intolerance (GTT) and insulin resistance (ITT) compared with AAV‐hSyn‐GFP mice (Figure 6J). Liver TAG levels were significantly lower in AAV‐hSyn‐Cre mice relative to AAV‐hSyn‐GFP mice 11 weeks after AAV transduction (Figure 6K). Liver lipid droplets were smaller and less abundant in AAV‐hSyn‐Cre mice (Figure S7D, Supporting Information). In light of these findings, we propose that PVH^SH2B1^ neurons sense/integrate, via SH2B1 pathways, neuronal and hormonal signals and relay metabolic information to the DRN, thereby shaping the ability of DRN neurons to control energy balance, body weight, and metabolism (Figure 6L).

### 2.10 SH2B1 Enhances BDNF Signaling in PVH^SH2B1^ Neurons

**Figure 7:**
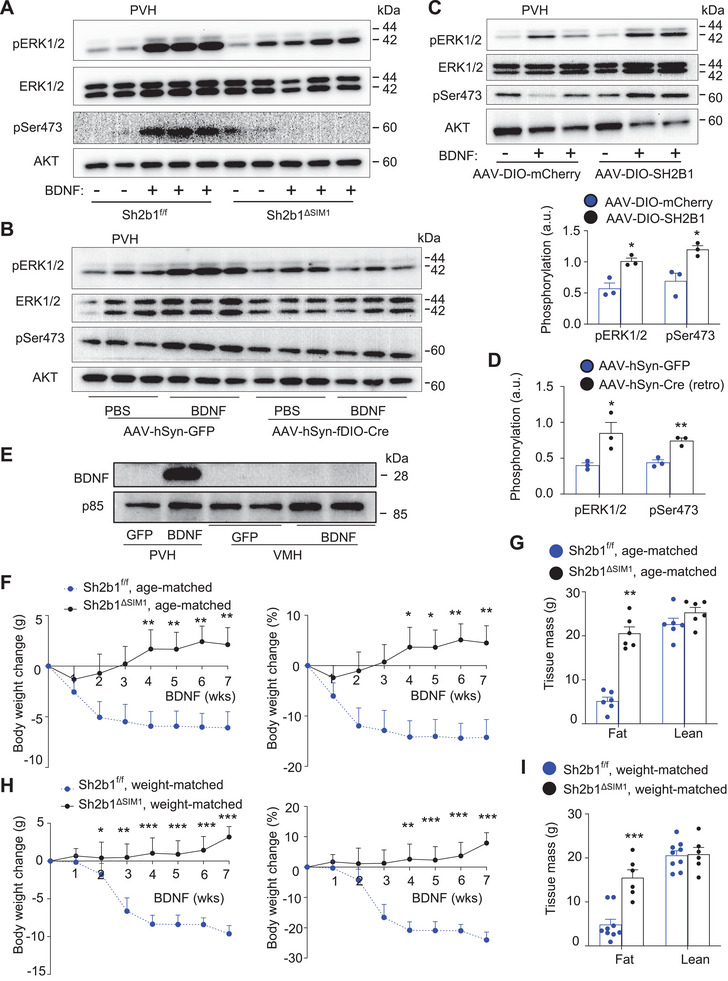
SH2B1 enhances BDNF action in the PVH. A) Males (18 weeks old) were stimulated with BDNF (60 ng per mouse, icv) for 20 min. PVH extracts were immunoblotted with the indicated antibodies. B) Sh2b1*^f/f^* males were bilaterally injected into the PVH with AAV8‐hSyn‐fDIO‐Cre or AAV‐GFP while AAV‐EF1a‐Flpo vector was injected into the DRN. Ten weeks later, mice were stimulated with BDNF (40 ng per mouse, icv) for 20 min. PVH extracts were immunoblotted with the indicated antibodies. C) Sh2b1*‐Cre* males were bilaterally microinjected into the PVH with AAV9‐CAG‐DIO‐SH2B1 or AAV‐CAG‐mCherry vector, fed HFD for 10 weeks, and stimulated with BDNF (20 ng per mouse, icv). PVH extracts were immunoblotted with the indicated antibodies. BDNF‐stimulated phosphorylation of ERK1/2 and AKT was normalized to total ERK1/2 and AKT, respectively (*n* = 3 mice per group). D) C57BL/6J males were microinjected into the PVH with AAV9‐CAG‐DIO‐SH2B1 bilaterally while retrograde AAV‐hSyn‐Cre or AAV‐hSyn‐GFP vector was injected into the DRN. Ten weeks later (on the HFD diet), they were stimulated with BDNF (20 ng per mouse, icv) for 20 min. ERK1/2 and AKT phosphorylation in PVH extracts were measured and normalized to total ERK1/2 and AKT, respectively (*n* = 3 mice per group). E–G) AAV9‐CAG‐proBDNF vectors were bilaterally injected into the PVH in males at 18 weeks of age (on a chow diet). E) PVH and VMH extracts were immunoblotted with antibodies to BDNF or p85 (loading control). F) Body weight changes after AAV transduction (six mice per group). G) Total fat and lean mass 5 weeks after AAV transduction (six mice per group). H, I) Sh2b1*^f/f^* males were fed an HFD for 6 weeks. Body weight‐matched Sh2b1*^f/f^* (on HFD) and Sh2b1*^ΔSIM1^* (on chow diet) males were bilaterally microinjected with AAV9‐CAG‐proBDNF vectors into the PVH. H) Body weight changes after AAV transduction. Sh2b1*^f/f^*: *n* = 9, Sh2b1*^ΔSIM1^*: *n* = 6. I) Total fat mass lean mass 5 weeks after AAV transduction. Sh2b1*^f/f^*: *n* = 9, Sh2b1*^ΔSIM1^*: *n* = 6. Data are presented as mean ± SEM. *p < 0.05 **p < 0.01, ***p < 0.001, C, D, G, I) 2‐tailed unpaired Student's *t‐*test F, H) or two‐way ANOVA.

To show that PVH neurons express both SH2B1 and BDNF receptor TrkB, hypothalamic sections were prepared from Sh2b1*‐Cre* mice to assess the expression of Cre (a surrogate for endogenous SH2B1) and TrkB by RNAscope. In the PVH, ≈80% of TrkB neurons (total 1658 TrkB^+^ neurons) also expressed SH2B1 (Cre^+^TrkB^+^) (Figure S8A, Supporting Information). To examine TrkB signaling, Sh2b1*^ΔSIM1^*, and Sh2b1*^f/f^* mice were stimulated with BDNF for 20 min. BDNF stimulated phosphorylation of ERK1/2 and AKT in PVH extracts in Sh2b1*^f/f^* but not Sh2b1*^ΔSIM1^* mice (**Figure** **7**A). ERK1/2 phosphorylation in the ventromedial hypothalamus (VMH) was comparable between Sh2b1*^f/f^* and Sh2b1*^ΔSIM1^* mice (Figure S8B, Supporting Information). To examine DRN‐projecting PVH^SH2B1^ neurons, Sh2b1*^f/f^* males were bilaterally microinjected into the PVH with AAV8‐hSyn‐fDIO‐Cre or AAV8‐hSyn‐GFP vector while retrograde AAV‐EF1a‐Flpo vector was microinjected into the DRN. Ten weeks later, mice were stimulated with BDNF for 20 min. BDNF‐stimulated phosphorylation of ERK1/2 and AKT in the PVH was substantially lower in AAV8‐hSyn‐fDIO‐Cre (deleting Sh2b1 in DRN‐projecting PVH^SH2B1^ neurons) than in AAV‐hSyn‐GFP mice (Figure 7B; Figure S8C, Supporting Information). To test if overexpression of SH2B1 enhances BDNF signaling, Sh2b1*‐Cre* males were bilaterally microinjected into the PVH with AAV9‐CAG‐DIO‐SH2B1 or AAV9‐CAG‐DIO‐mCherry (control) vector. Ten weeks later, mice were stimulated with BDNF (20 ng/mouse, icv) for 20 min. BDNF‐stimulated phosphorylation of ERK1/2 and AKT in the PVH was markedly higher in AAV9‐CAG‐DIO‐SH2B1 than in AAV9‐CAG‐DIO‐mCherry mice (Figure 7C). Phosphorylation of ERK1/2 and AKT in the VMH was comparable between the two groups (Figure S8D, Supporting Information). To test if overexpression of SH2B1 in the DRN‐projecting subpopulation also enhances BDNF signaling, C57BL/6 males were bilaterally microinjected into the PVH with AAV9‐CAG‐DIO‐SH2B1 vector while retrograde AAV‐hSyn‐Cre or AAV‐hSyn‐GFP (control) vector was injected into the DRN. Mice were stimulated with BDNF 11 weeks later. BDNF‐stimulated phosphorylation of ERK1/2 and AKT in the PVH was significantly higher in AAV‐hSyn‐Cre (SH2B1 overexpression in DRN‐projecting PVH neurons) than in AAV‐hSyn‐GFP mice (Figure 7D; Figure S8E, Supporting Information).

### 2.11 PVH^SH2B1^ Neuron‐Intrinsic SH2B1 Mediates the Anti‐Obesity Action of PVH BDNF

To deliver BDNF to the PVH, we generated an AAV‐CAG‐proBDNF vector (encoding the full length of mouse BDNF). We validated the vector and BDNF expression/secretion in N2a culture as well as in mouse brains (Figure S9A,B, Supporting Information). AAV9‐CAG‐proBDNF vector was bilaterally microinjected into the PVH of Sh2b1*^ΔSIM1^* or Sh2b1*^f/f^* males to chronically deliver BDNF to the PVH. We confirmed that BDNF overexpression was restricted to the PVH and was not spread to the VMH (Figure S9A, Supporting Information). Endogenous BDNF was below the detection threshold. BDNF caused weight loss in Sh2b1*^f/f^* but not Sh2b1*^ΔSIM1^* mice (Figure S9B,C, Supporting Information). Total fat mass, but not lean mass, was significantly higher in Sh2b1*^ΔSIM1^* than in Sh2b1*^f/f^* mice 5 weeks after BDNF expression (Figure 7G). Glucose intolerance and insulin resistance were more severe in Sh2b1*^ΔSIM1^* than in Sh2b1*^f/f^* mice after BDNF expression (Figure S9D, Supporting Information).

Notably, Sh2b1*^ΔSIM1^* mice were obese before AAV9‐CAG‐proBDNF transduction (Figure S9C, Supporting Information). Obesity might influence BDNF response. To address this concern, Sh2b1*^f/f^* mice were fed an HFD for 6 weeks to increase body weight, whereas Sh2b1*^ΔSIM1^* mice were fed a normal chow diet. Body weight‐matched Sh2b1*^f/f^* and Sh2b1*^ΔSIM1^* mice were bilaterally microinjected with AAV9‐CAG‐proBDNF vector into the PVH. Consistently, BDNF stimulated weight loss in Sh2b1*^f/f^* but not Sh2b1*^ΔSIM1^* mice (Figure 7H; Figure S9E, Supporting Information). Total fat mass, but not lean mass, was significantly lower in Sh2b1*^f/f^* than in Sh2b1*^ΔSIM1^* mice 5 weeks post AAV9‐CAG‐proBDNF transduction (Figure 7I). Sh2b1*^ΔSIM1^* mice developed more severe glucose intolerance and insulin resistance after AAV9‐CAG‐proBDNF transduction (Figure S9F, Supporting Information). Collectively, these results suggest that PVH^SH2B1^ neuron‐intrinsic SH2B1 protects against energy imbalance and obesity at least in part by enhancing BDNF/TrkB pathways in the PVH (Figure 6L).

## 3 Discussion

The PVH encompasses heterogeneous neuronal populations projecting to broad brain areas. Distinct PVH neurocircuits play unique and important roles in controlling energy balance, metabolism, and body weight. In this work, we mapped a previously unrecognized PVH^SH2B1^→DRN neurocircuit that pivotally controls body weight and metabolism (Figure 6L). The DRN emerges as an important energy balance center.^[^ ^15^ ^]^ PVH^SH2B1^ neurons sent axons to the DRN and formed monosynaptic connections with DRN neurons. Target neuron phenotypes (e.g., DRN^5‐HT^, DRN^Vglut3^, and/or DRN^Vgat^) are currently unknown. Optogenetic stimulation of PVH^SH2B1^ axonal fibers in the DRN rapidly suppressed feeding behavior. Chronic inhibition of PVH^SH2B1^ neurons causes obesity, glucose intolerance, insulin resistance, and MASLD. Considering that DRN^5‐HT^ and DRN^Vglut3^ neurons suppress appetite, we are tempted to propose that PVH^SH2B1^ neurons directly activate anorexigenic DRN^5‐HT^ and/or DRN^Vglut3^ neurons, thereby inhibiting food intake. In line with this notion, glutamatergic neurons were reported to be predominant in the PVH.^[^ ^7^, ^21^ ^]^ Importantly, glutamatergic neurons accounted for a large portion of DRN‐projecting PVH^SH2B1^ neurons. Additionally, PVH^SH2B1^ neurons may also stimulate DRN^5‐HT^ and/or DRN^Vglut3^ neurons indirectly through local interneurons in the DRN.

We demonstrated that PVH^SH2B1^ neuron‐intrinsic BDNF/TrkB/SH2B1 pathways safeguard the function of the PVH^SH2B1^→DRN neurocircuit. Both embryonic‐onset and adult‐onset deletion of Sh2b1 in PVH neurons caused hyperphagia, reduced energy expenditure, obesity, insulin resistance, glucose intolerance, and MASLD. Likewise, deletion of Sh2b1 in DRN‐projecting PVH^SH2B1^ neurons, using the Cre‐dependent, Flp‐dependent, and retrograde AAV paradigm, also caused energy imbalance, obesity, insulin resistance, and MASLD. Conversely, overexpression of SH2B1 in either total or DRN‐projecting PVH^SH2B1^ neurons protected against HFD‐induced obesity, metabolic disorders, and MASLD. Thus, SH2B1 levels in PVH^SH2B1^ neurons critically influence body weight, raising the possibility that SH2B1 may act as a molecular rheostat to control energy balance and metabolism. These findings point to a new anti‐obesity strategy by upregulating or activating hypothalamic SH2B1.

Given that LepRb‐expressing neurons are sparse in the PVH, it is unlikely that SH2B1 in the PVH protects against obesity by directly enhancing leptin signaling. We found that deletion of Sh2b1 in either total or DRN‐projecting PVH^SH2B1^ neurons inhibited BDNF signaling in the PVH. Conversely, overexpression of SH2B1 enhanced PVH BDNF signaling. In line with these results, SH2B1 directly binds, via its SH2 domain, to TrkB to enhance BDNF signaling in cell culture.^[^ ^8g^ ^]^ Remarkably, ablation of Sh2b1 in PVH^SH2B1^ neurons abrogated the ability of PVH BDNF to protect against hyperphagia, obesity, and metabolic disorders. Collectively, these results support a model that PVH^SH2B1^ neuron‐intrinsic BDNF/TrkB/SH2B1 pathways safeguard the activation and function of the PVH^SH2B1^→DRN neurocircuit (Figure 6L). Thus, the PVH^SH2B1^→DRN neurocircuit may serve as a new paradigm to investigate brain control of body weight and metabolism.

## 4 Experimental Section

### Animals

Sh2b1*^f/f,^* Sh2b1*‐Cre*, and *Sim1‐Cre* mice (C57BL/6 background) were characterized previously.^[^ ^6^, ^8^, ^12^, ^19^ ^]^ Mice were housed on a 12 h light‐dark cycle at 25 °C and fed ad libitum a normal chow diet (9% fat; TestDiet, St. Louis, MO) or a HFD (60% fat; Research Diets, New Brunswick, NJ).

### Ethics Statements

Animal research complied with all relevant ethnic regulations. Animal experiments were conducted following the protocol PRO00011480 approved by the University of Michigan Institutional Animal Care and Use Committee (IACUC).

### AAV Vectors

Human SH2B1*β* cDNA sequences were double‐flanked by two identical loxp sequences in a head‐to‐head orientation, and the double‐floxed SH2B1*β* cDNA in an inverse orientation (DIO) was inserted into the 3′ end of the *CAG* promoter (AAV‐CAG‐DIO‐SH2B1). *Cre* cDNA sequences were double‐flanked by two identical FRT sequences in a head‐to‐head orientation. Double‐floxed *Cre* cDNA in an inverse orientation (fDIO) was inserted into the 3′ end of the *hSyn* promoter (AAV‐hSyn‐fDIO‐Cre). Murine *pro‐Bdnf* cDNA, which encodes the full length of BDNF (NM_001285416.1), was inserted into an AAV‐CAG‐proBDNF vector. BDNF expression is under the control of the *CAG* promoter. AAV9‐CAG‐DIO‐SH2B1 (1.18 × 10^11^ vg mL^−1^), AAV9‐CAG‐proBDNF (2.8 × 10^12^ vg mL^−1^), AAV9‐hSyn‐DIO‐Tettox (2.7 × 10^12^ vg mL^−1^), AAV8‐hSyn‐fDIO‐Cre (1.3 × 10^13^ vg mL^−1^), and AAV9‐CAG‐DIO‐mCherry (4.6 × 10^12^ vg mL^−1^) vectors were prepared in the lab. Following vectors were purchased from the Addgene: retrograde AAV‐hSyn‐Cre (107738‐AAVrg, 2.3 × 10^13^ vg mL^−1^), retrograde AAV‐hSyn‐DIO‐mCherry (4 × 10^12^ vg mL^−1^), retrograde AAV‐EF1a‐Flpo (55637‐AAVrg, 1.6 × 10^13^ vg mL^−1^), AAV1‐hSyn‐DIO‐mGFP‐2A‐Synaptophysin‐mRuby (71760‐AAV1, 2.7 × 10^13^ vg mL^−1^), AAV5‐EF1α‐DIO‐hChR2(H134R)‐EYFP (20298, 1.1 × 10^13^ vg mL^−1^), AAV5‐EF1α‐DIO‐ EYFP (27056, 1.3 × 10^13^ vg mL^−1^), and AAV9‐hSyn‐DIO‐mCherry (50459, 1 × 10^12^ vg mL^−1^). AAV1‐hSyn‐Cre (2.3 × 10^13^ vg mL^−1^) and AAV1‐hSyn‐GFP (1.2 × 10^13^ vg mL^−1^) vectors were purchased from the Vector Core at the University of Pennsylvania School of Medicine.

### Stereotaxic Microinjection

Male mice were isoflurane‐anesthetized and mounted on an Ultra Precise Small Animal Stereotaxic Alignment System (David KOPF Instruments, Tujunga, CA). A small opening was made in the skull. AAV vectors (0.3 µL for PVH and 1 µL for DRN) were bilaterally or unilaterally microinjected into the PVH (mm: −0.85 a‐p, ±0.15 m‐l, −5.1 d‐v) or DRN (mm: −4.75 a‐p, 0 m‐l, −3.33 d‐v), using UltraMicroPumps with SYS‐Micro4 Controller (UMP3‐2, World Precision Instruments Inc, Sarasota, FL).

### GTT and ITT

For GTT, mice were fasted overnight and intraperitoneally injected with glucose (2 g kg^−1^ body weight). For ITT, mice were fasted for 6 h and intraperitoneally injected with insulin (0.75 units kg^−1^). Blood glucose was measured 0, 15, 30, 60, and 120 min after injection.

### Fat Content and Energy Expenditure

Fat content and lean body mass (normalized to body weight) were measured using a dual‐energy X‐ray absorptiometry pDexa (Norland Stratec). Oxygen consumption (VO_2_), carbon dioxide production (VCO_2_), and food intake were measured at 20–23 °C and 12‐12 h dark‐light cycles with free access to food and water, using Comprehensive Laboratory Monitoring System (CLAMS, Columbus Instruments) in the University of Michigan Metabolic, Physiological and Behavioral Core. Energy expenditure was analyzed using ANCOVA as described by the National Mouse Metabolic Phenotying Center (MMPC): http://www.mmpc.org/shared/regression.aspx.

### Liver TAG Levels

Liver samples were homogenized in 1% acetic acid and extracted by chloroform:methanol (2:1). The organic phase was dried by evaporation and dissolved in isopropanol. TAG levels were measured using a TAG assay kit (Pointe Scientific Inc., Canton, MI), and normalized to liver weight.

### Immunostaining, Immunoprecipitation, and Immunoblotting

Frozen brain, BAT, and liver sections were cut using Leica cryostat (Leica Biosystems Nussloch GmbH, Nussloch, Germany), and immunostained with appropriate antibodies (Table S1, Supporting Information). Images were obtained, using BX51 Microscope coupled with a DP72 digital camera (Olympus, Tokyo, Japan). H&E staining of WAT, BAT, and liver samples was performed on paraffin sections. Tissues or cell cultures were homogenized in ice‐cold lysis buffer (50 mm Tris HCl, pH 7.5, 0.5% Nonidet P‐40, 150 mm NaCl, 2 mm EGTA, 1 mm Na_3_VO_4_, 100 mm NaF, 10 mm Na_4_P_2_O_7_, 1 mm phenylmethylsulfonyl fluoride, 10 µg mL^−1^ aprotinin, 10 µg mL^−1^ leupeptin). Tissue or cell extracts were immunoprecipitated and immunoblotted with appropriate antibodies (Table S1, Supporting Information).

### qPCR

Total RNAs were extracted from cells and tissue samples using TRIzol reagent (Invitrogen Life Technologies, Carlsbad, CA). The first‐strand cDNAs were synthesized using random primers and M‐MLV reverse transcriptase (Promega, Madison, WI). qPCR was performed, using Radiant SYBR Green 2× Lo‐ROX qPCR Kits (Alkali Scientific, Pompano Beach, FL), a StepOnePlus RT PCR Systems (Life Technologies Corporation, NY, USA), and appropriate primers (Table S2, Supporting Information).

### Optogenetic Stimulation

Adult Sh2b1*‐Cre* male mice were anesthetized with isoflurane (5% for induction, 1.5% for maintenance) and injected with 5 mg kg^−1^ of carprofen before being placed in a stereotactic apparatus. Body temperature was maintained at 35–37 °C. Bilateral injections of AAV5‐EF1α‐DIO‐hChR2(H134R)‐EYFP (200 nL, 2.2 × 10^13^ GC mL^−1^) were administered into the PVH (0.7 mm posterior, 0.2 mm lateral, 4.7 mm ventral to bregma). AAV5‐EF1α‐DIO‐EYFP (200 nL, 1.3 × 10^13^ GC mL^−1^) was used as a control. An optic fiber (200 µm core, 0.22 NA) was implanted above the DRN (4.75 mm posterior, 0 mm lateral, and 3.15 mm ventral to bregma). After 4 weeks of recovery, mice were singly housed in their home cages and acclimated. Mice were fasted overnight and then given regular chow the following morning. Food intake in the first hour was measured when the PVH ^SH2B1^→DRN circuit was photostimulated (473 nm, 10 ms per pulse, 20 Hz) or when no light was given (control).

### Circuit Mapping

AAV1‐hSyn‐FLEX‐mGFP‐2A‐Synaptophysin‐mRuby vector (100 nL, 2.7 × 10^13^ GC mL^−1^) were unilaterally injected into the PVH (0.7 mm posterior, 0.2 mm lateral, and 4.7 mm ventral to bregma) of Sh2b1*‐Cre* male. After a 4‐week recovery period, the animals were euthanized and transcardially perfused with phosphate‐buffered saline (PBS) and 4% paraformaldehyde (PFA). The brain was then harvested, post‐fixed overnight in 4% PFA, and cryoprotected in 30% sucrose for 48 h at 4 °C. The brain‐frozen sections (40 µm) were mounted onto glass slides, stained with DAPI, and then mounted with Prolong Gold mounting media (Invitrogen, #P36930). Histological images (DAPI, GFP, and mRuby) were acquired using a Nikon A1 Confocal microscope.

### RNAscope

Retrograde AAV‐DIO‐mCherry vectors were injected into the DRN of Sh2b1*‐Cre* male (4.75 mm posterior, 0 mm lateral, and 3.3 mm ventral to bregma). 4 weeks later, mice were anesthetized with isoflurane, and the brain was rapidly isolated, and embedded into a pre‐chilled optimum cutting temperature compound (OCT). The OCT block was quickly placed in dry ice for rapid freezing, and brain sections (16 µm thickness) were prepared using a Leica cryostat CM3050 S cryostat. The sections were fixed in 4% PFA, dehydrated through an ethanol series, and treated with Pretreatment Reagent (Advanced Cell Diagnostics). RNAscope Multiplex Fluorescent in situ assay Reagent Kit v2 Assay was performed using proprietary RNAscope technology with their probes: *Slc17a6* (Ref: 456751‐C2; Lot: 23151B), *mCherry* (Ref: 431201‐C4; Lot: 23072B), *Slc32a1* (Ref: 319191‐C3; Lot: 23151B), *Cre* (Ref: 312281‐C4; Lot: 24054), and *TrkB* (Ref: 423611‐C3; Lot: 24052C). Sections were mounted on slides with prolonged gold antifade reagent (Invitrogen; P36930) and imaged using a Nikon A1 confocal microscope. The sections were compared to the brain atlas to localize the PVH. PVH mCherry^+^, Vglut2^+^ (Slc17a6), Vgat^+^ (Slc32a1), Cre^+^, and TrkB^+^ neurons were counted.

### Statistical Analysis

Data were presented as means ± SEM. Differences between the two groups were analyzed by a 2‐tailed Student's *t*‐test. Comparisons between more than two groups/variables were analyzed by one‐way or two‐way ANOVA/Tukey's post hoc test using GraphPad Prism 8. A P value less than 0.05 was considered significant.

## Conflict of Interest

The authors declare no conflict of interest.

## Author Contributions

Y.L., L.J., L.B., X.L., and N.G. conducted experiments, Y.L. and L.R. designed experiments and wrote the paper, L.D.F. and D.P.O. provided AAV8‐hSyn‐fDIO‐Cre, and Y.L., M.H.K., D.P.O., P.L., and L.R. edited the paper.

## Supporting information

Supporting Information

## Data Availability

The data that support the findings of this study are available in the supplementary material of this article.
