# Supplementary material for: SH2B1 Defends Against Energy Imbalance, Obesity, and Metabolic Disease via a Paraventricular Hypothalamus→Dorsal Raphe Nucleus Neurocircuit
Source: Adv Sci (Weinh). 2024 Jun 17;11(31):2400437. doi: 10.1002/advs.202400437 (PMC11336965; doi:10.1002/advs.202400437)
Supplement: Supplementary file 1 — Supporting Information [file ADVS-11-2400437-s001.pdf]

ADVANCED SCIENCE
Open Access

Supporting Information

for *Adv. Sci.*, DOI 10.1002/advs.202400437

SH2B1 Defends Against Energy Imbalance, Obesity, and Metabolic Disease via a Paraventricular Hypothalamus→Dorsal Raphe Nucleus Neurocircuit

*Yuan Li, Min-Hyun Kim, Lin Jiang, Lorelei Baron, Latrice D. Faulkner, David P. Olson, Xingyu Li, Noam Gannot, Peng Li and Liangyou Rui**

Supporting Information

SH2B1 DEFENDS AGAINST ENERGY IMBALANCE, OBESITY, AND METABOLIC DISEASE VIA A PARAVENTRICULAR HYPOTHALAMUS→DORSAL RAPHE NUCLEUS NEUROCIRCUIT

Yuan Li[1], Min-Hyun Kim[1,2], Lin Jiang[1], Lorelei Baron[1], David P Olson[1,3,4], Xingyu Li[5], Noam Gannot[5,6], Peng Li[1,5,6], Liangyou Rui[1,4,7*]

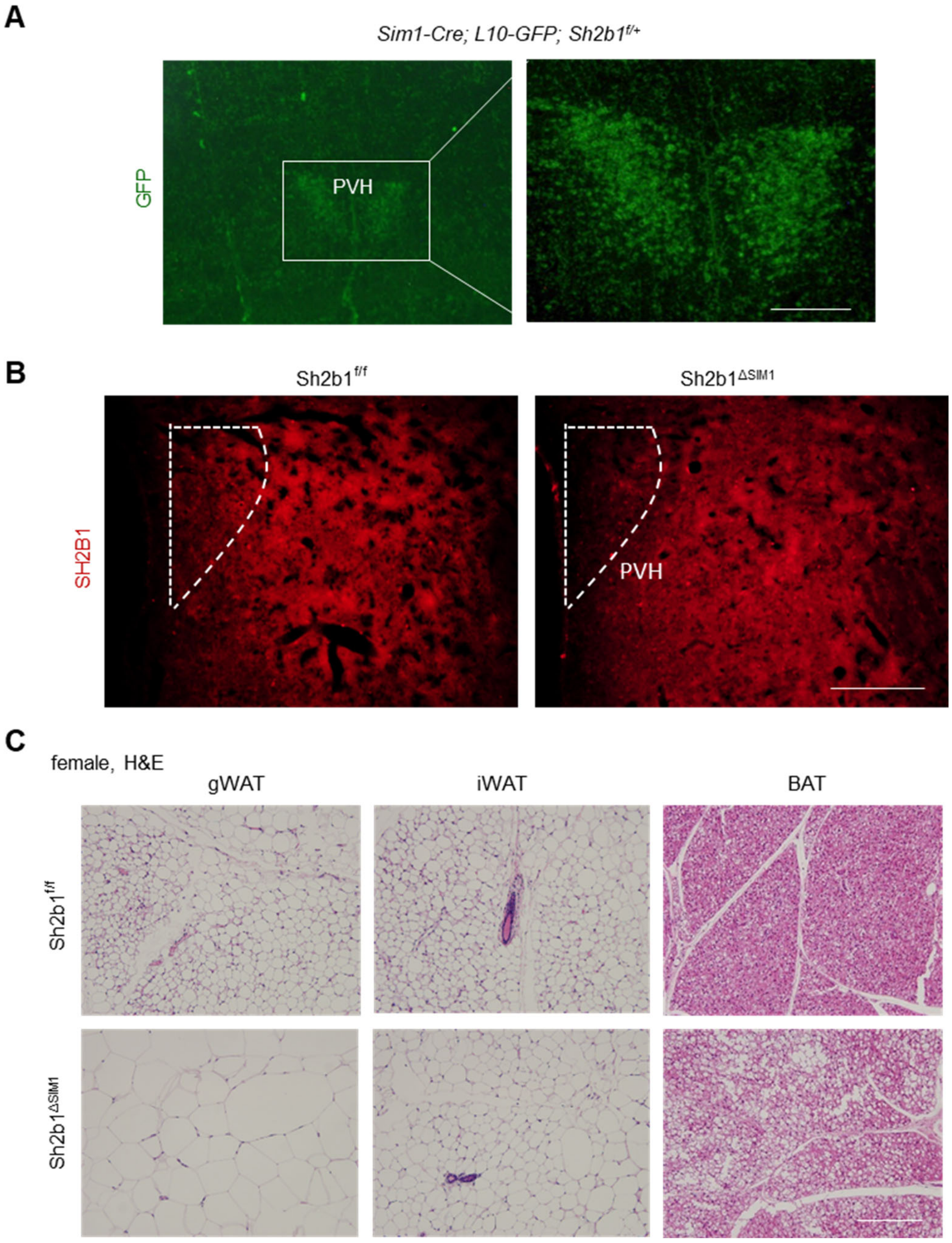

Figure S1. SIM1 neuron-specific deletion of *Sh2b1* induces white adipocyte hypertrophy and BAT whitening. (A) Hypothalamic sections were obtained from *Sh2b1$^{f/f}$;L10-GFP;Sim1-Cre* mice. The PVH was demarcated by GFP expression. Scale bar: 200 μm. **(B)** Hypothalamic sections were prepared from males at 10 weeks of age and stained with anti-SH2B1 antibody (Santa Cruz sc-136065). **(C)** H&E staining of WAT, BAT, and liver sections from female mice at 18 weeks of age on normal chow diet (3 mice per group).

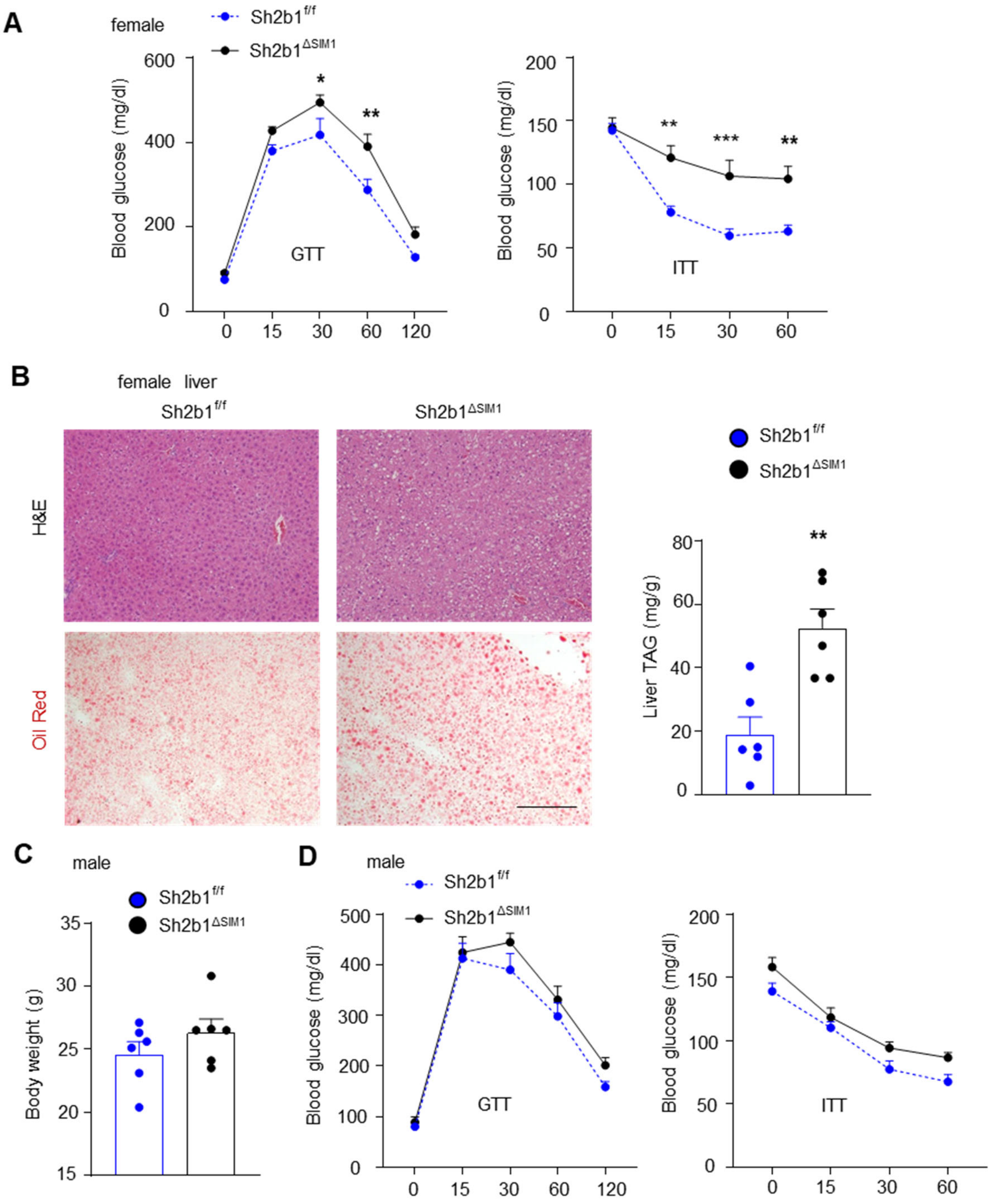

Figure S2. SIM1 neuron-specific deletion of *Sh2b1* induces glucose intolerance, insulin resistance, and liver steatosis in female mice. (A) GTT and ITT were performed in chow-fed female mice at 18 weeks of age. *Sh2b1*$^{f/f}$: n=6 for GTT and n=8 for ITT, *Sh2b1*$^{\Delta SIM1}$: n=8 for GTT and n=7 for ITT. **(B)** H&E and Oil Red O staining of liver sections (3 mice per group). Scale bar: 200 µm. **(C)** Male body weight at 8 weeks of age (on normal chow diet). **(D)** GTT and ITT in males at 8 weeks of age (on chow diet). *Sh2b1*$^{f/f}$: n=6, *Sh2b1*$^{\Delta SIM1}$: n=6. Data are presented as mean ± SEM. *p<0.05, **p<0.01, ***p<0.001, 2-tailed unpaired Student's *t* test (C) or two-way ANOVA (A, D).

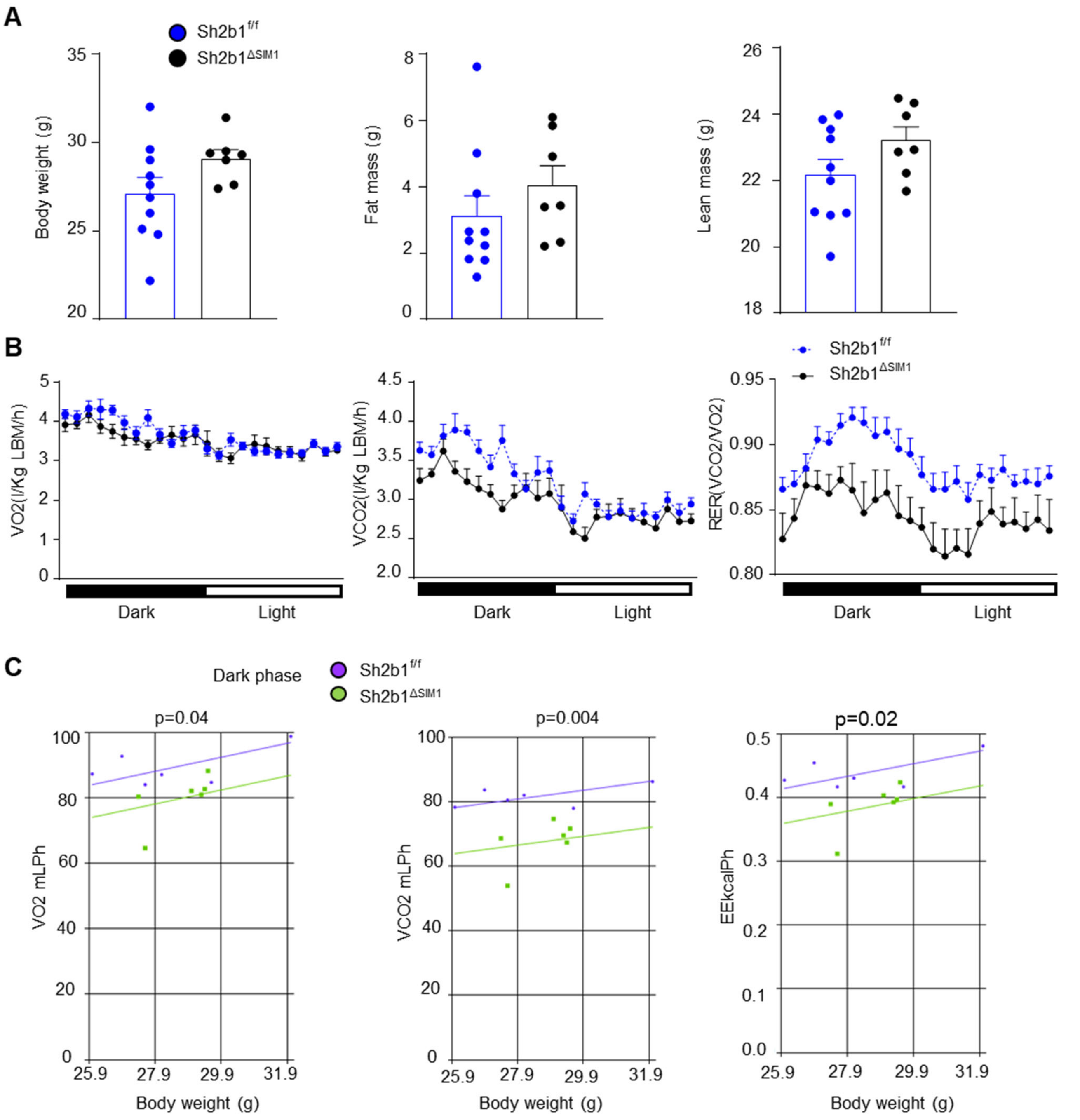

Figure S3. SIM1 neuron-specific deletion of *Sh2b1* decreases energy expenditure. Male mice were fed a normal chow diet and subjected to CLAMS at 9 weeks of age. **(A)** Body weight, fat content, and lean mass. *Sh2b1^f/f^*: n=10, *Sh2b1^ΔSIM1^*: n=7. **(B)** VO_2 and VCO_2 values were normalized to lean mass. *Sh2b1^f/f^*: n=10, *Sh2b1^ΔSIM1^*: n=7. **(C)** Analysis of covariance (ANCOVA). *Sh2b1^f/f^*: n=6, *Sh2b1^ΔSIM1^*: n=6. Data are presented as mean ± SEM. *p<0.05, **p<0.01, ***p<0.001, 2-tailed unpaired Student's *t* test (A) or two-way ANOVA (B).

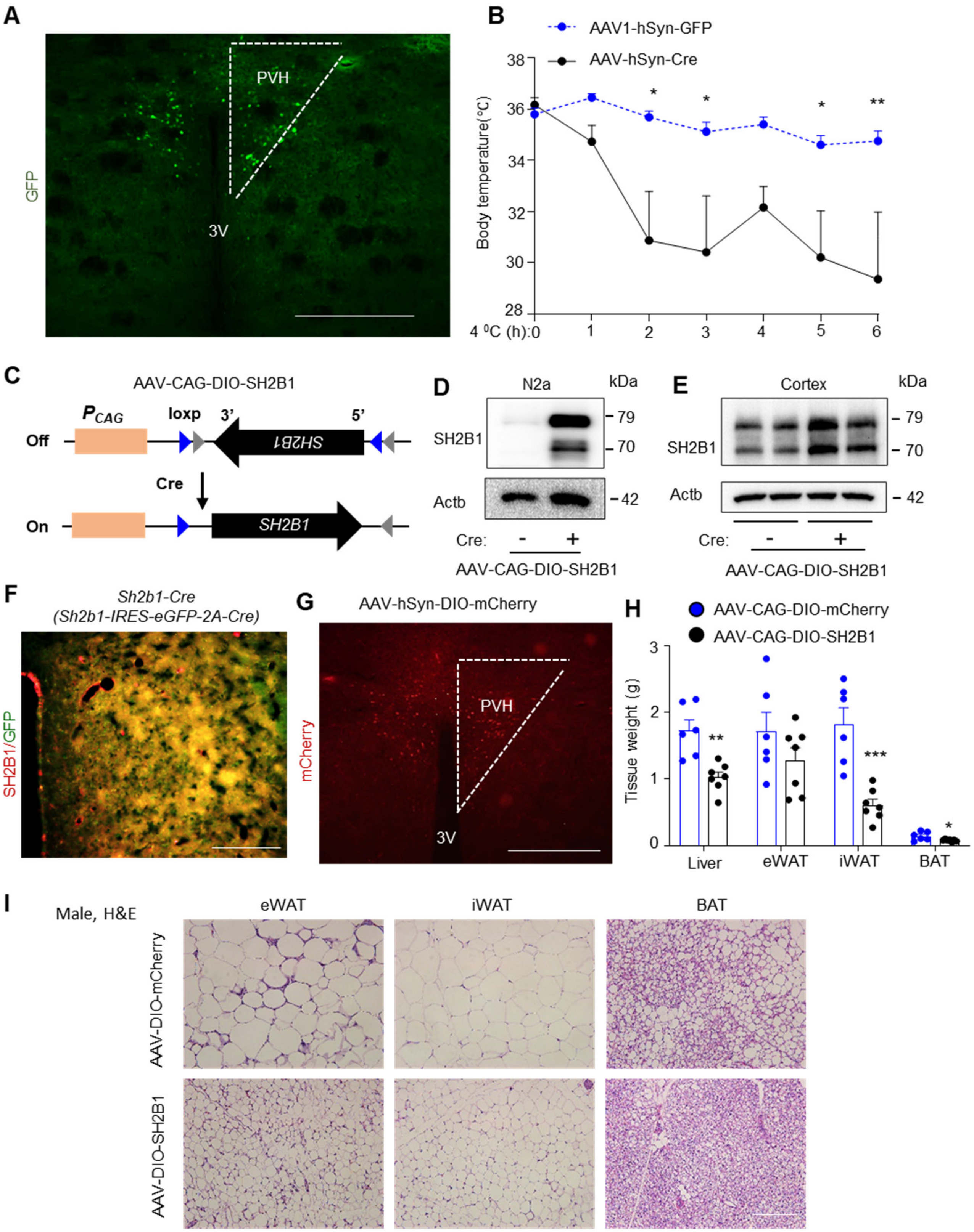

Figure S4. Effects of adult-onset ablation or overexpression of SH2B1 in the PVH on adipose thermogenesis. (A) AAV9-hSyn-GFP vectors were bilaterally microinjected into the PVH. The PVH was demarcated by GFP. Scale bar: 500 µm. **(B)** *Sh2b1*$^{f/f}$ males were bilaterally microinjected with AAV1-hSyn-Cre or AAV-hSyn-GFP vector into the PVH and14 weeks, they were subjected to cold

tolerance tests (4 ºC). AAV1-hSyn-GFP: n=7, AAV1-hSyn-Cre: n=6. **(C)** Schematic representation of AAV-CAG-DIO-SH2B1 vector. **(D)** AAV-CAG-DIO-SH2B1 plasmids were cotransfected into N2a cells with or without AAV-CMV-CreGFP plasmids. Cell extracts were prepared 48 h after transfection and immunoblotted with the indicated antibody. **(E)** AAV9-CAG-DIO-SH2B1 vector was co-microinjected with or without AAV-hSyn-Cre vectors into the cortex in mice. Three weeks later, cortical extracts were immunoblotted with the indicated antibodies. **(F)** Hypothalamic sections were prepared from *Sh2b1-Cre* males at 8 weeks and costained with anti-SH2B1 and anti-GFP antibodies. Scale bar: 200 µm. **(G-I)** *Sh2b1-Cre* males were bilaterally microinjected with AAV9-CAG-DIO-SH2B1 or AAV9-hSyn-DIO-mCherry vector into the PVH. **(G)** The PVH was demarcated by mCherry. Scale bar: 500 µm. **(H)** Tissue weight (10 weeks after AAV transduction). AAV-DIO-mCherry: n=6, AAV-DIO-SH2B1: n=7. **(I)** H&E staining of tissue sections. Scale bar: 200 µm. Data are presented as mean ± SEM. *p<0.05, **p<0.01, ***p<0.001, 2-tailed unpaired Student's *t* test (**H**) or two-way ANOVA (**B**).

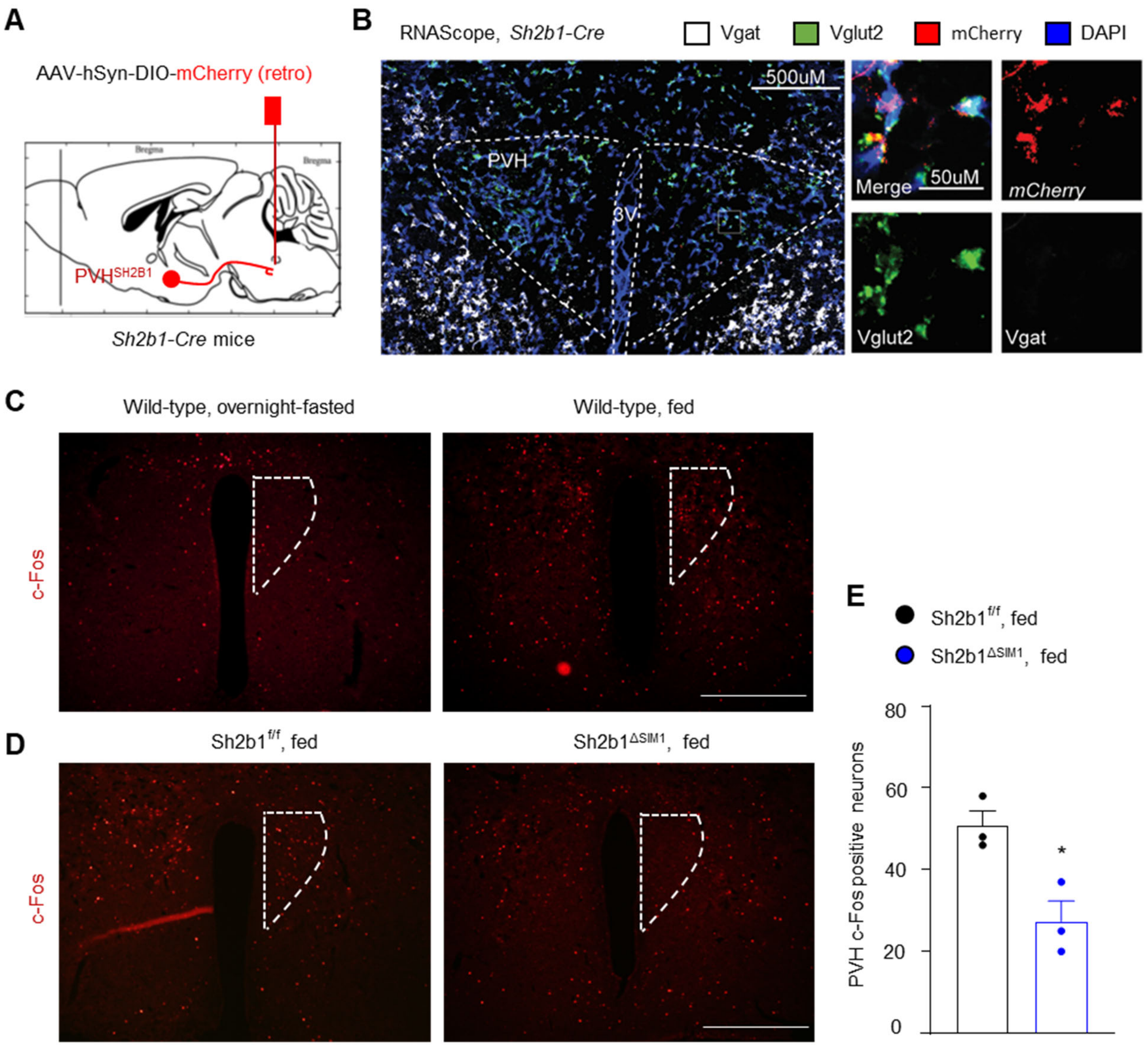

Figure S5. SH2B1 enhances PVH^{SH2B1} neuron activation under fed conditions. (A-B) Retrograde AAV-hSyn-DIO-mCherry vector was microinjected into the DRN in *Sh2b1-Cre* males (**A**). **(B)**

Hypothalamic sections were prepared 6 weeks after AAV transduction. Expression of mCherry, Vgat, and Vglut2 was measured in the PVH using RNAscope. **(C)** C57BL/6J males (10 weeks old) were fasted overnight or randomly fed. Hypothalamic sections were stained with anti-c-Fos antibody. Scale bar: 500 µm. **(D-E)** Hypothalamic sections were prepared from male mice (10 weeks old) and stained with anti-c-Fos antibody. c-Fos^{+} cells were counted in the PVH (n=3 per group). Scale bar: 500 µm. Data are presented as mean ± SEM. *p<0.05, 2-tailed unpaired Student's *t* test.

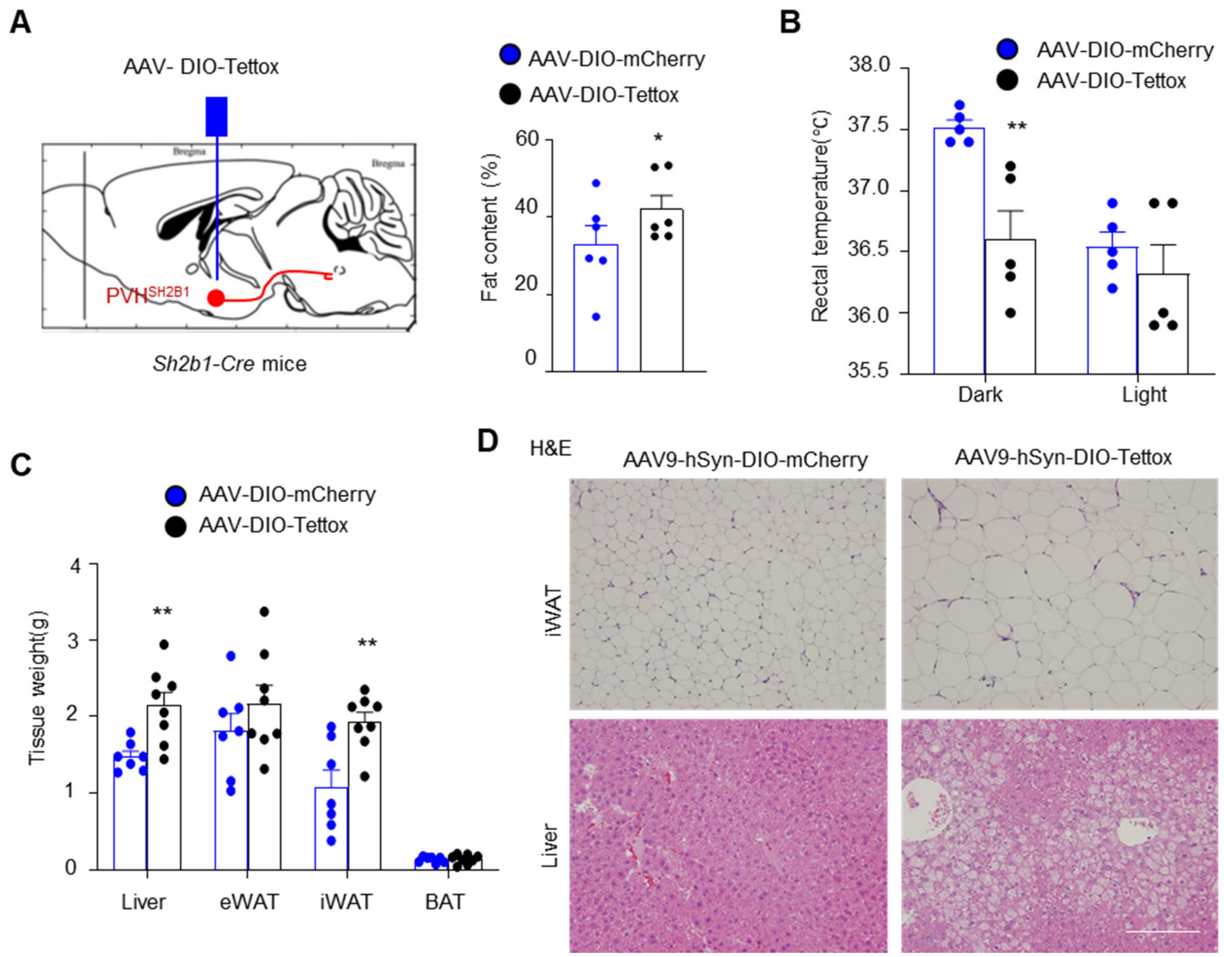

Figure S6. Inhibition of PVH^{SH2B1} neurons induces obesity and MASLD. AAV9-hSyn-DIO-Tettox or AAV9-hSyn-DIO-mCherry vectors were bilaterally microinjected into the PVH in *Sh2b1-Cre* males. **(A)** Fat content in 7 weeks after AAV transduction. AAV9-hSyn-DIO-mCherry: n=6, AAV9-hSyn-DIO-Tettox: n=6. **(B)** Rectal temperature in 7 weeks post AAV transduction. AAV9-hSyn-DIO-mCherry: n=5, AAV9-hSyn-DIO-Tettox: n=5. **(C)** Tissue weights 12 weeks post AAV transduction. AAV9-hSyn-DIO-mCherry: n=7, AAV9-hSyn-DIO-Tettox: n=8. **(D)** H&E staining of iWAT and liver sections 12 weeks post AAV transduction. Scale bar: 200 µm. Data are presented as mean ± SEM. *p<0.05, **p<0.01, 2-tailed unpaired Student's *t* test.

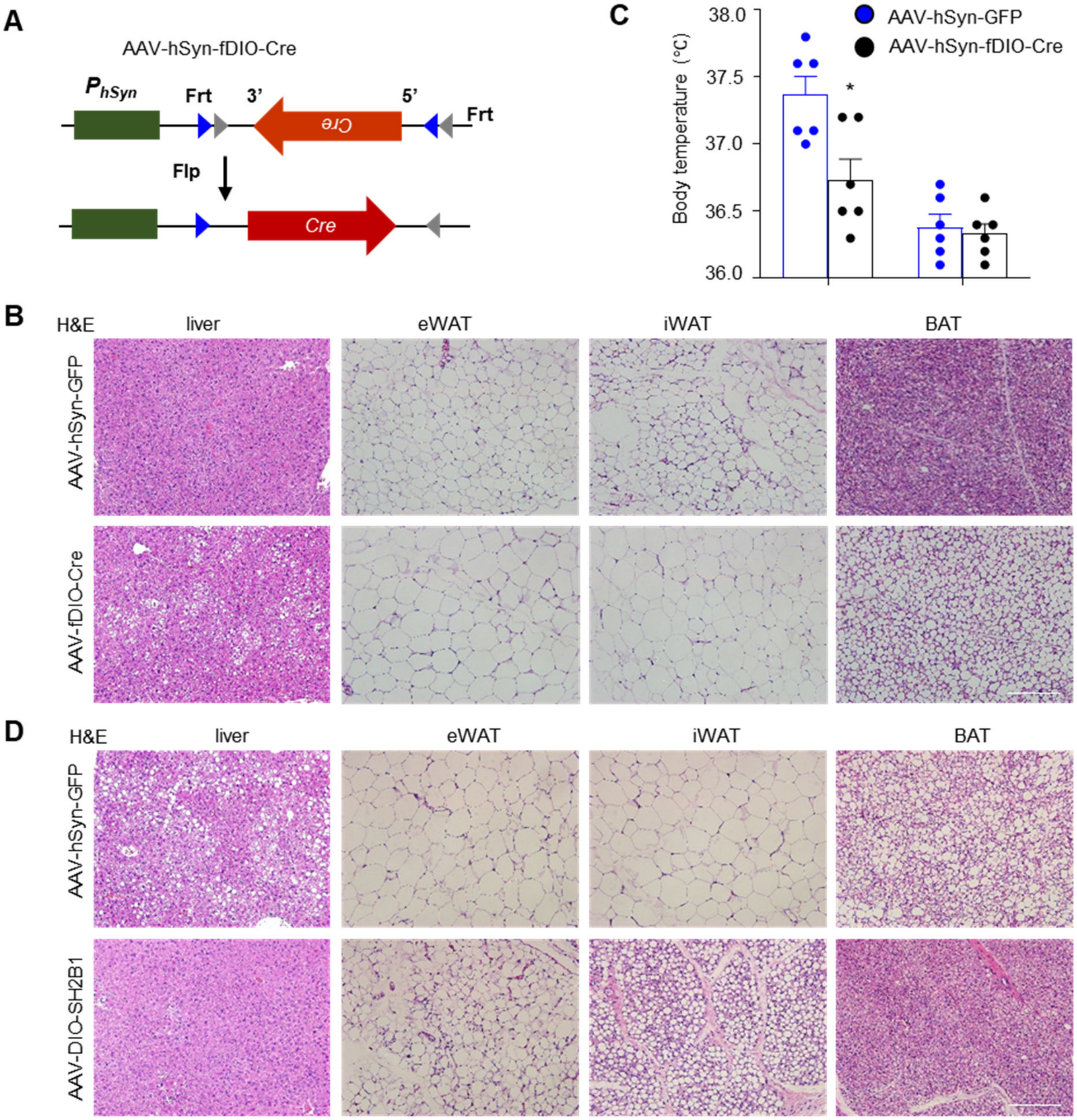

Figure S7. DRN-projecting PVH^{SH2B1} neuron-intrinsic SH2B1 defends against obesity and MASLD. (A) Schematic representation of AAV-hSyn-fDIO-Cre vectors. **(B-C)** *Sh2b1$^{f/f}$* males were bilaterally microinjected with AAV8-hSyn-fDIO-Cre or AAV-EF1a-GFP vector into the PVH and injected with retrograde AAV-EF1a-Flpo vector into and DRN (on normal chow diet). **(B)** H&E staining of WAT, BAT, and liver sections (10 weeks after AAV injection). **(C)** Rectal temperature 4 weeks post AAV transduction. **(D)** C57BL/6J males were bilaterally microinjected with AAV9-CAG-DIO-SH2B1 or AAV9-CAG-DIO-GFP into the PVH and with retrograde AAV-hSyn-Cre vector into the DRN. One week later, the mice were fed a HFD for 10 weeks. WAT, BAT, and liver sections were stained with H&E reagents. Data are presented as mean ± SEM. *p<0.05, 2-tailed unpaired Student's *t* test.

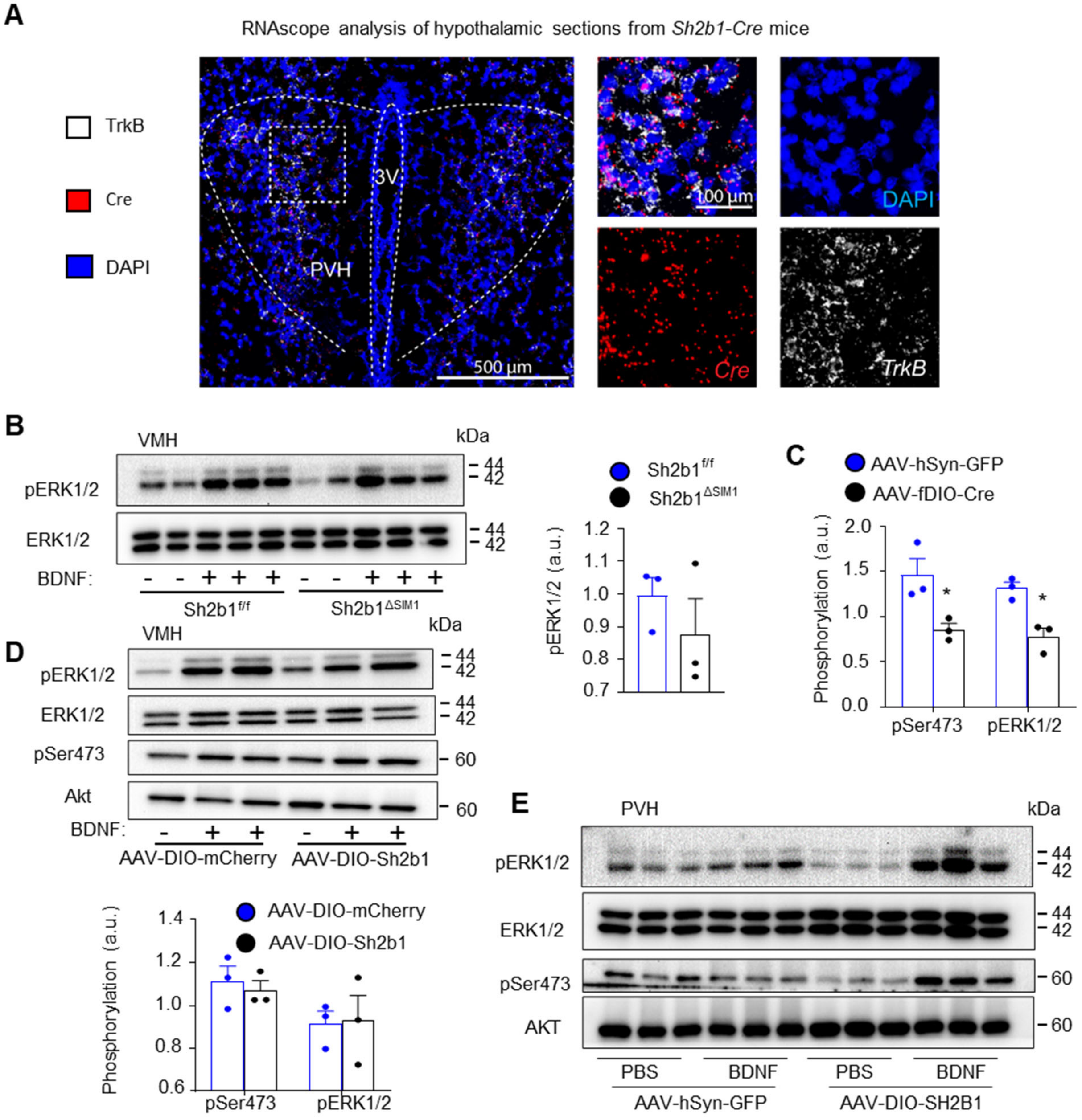

Figure S8. SH2B1 mediates BDNF signaling in the PVH. (A) Hypothalamic sections were prepared from Sh2b1-Cre males (10 weeks old) and used to perform a RNAscope analysis using *Cre* and *TrkB* probes (n=2 mice). **(B)** *$Sh2b1^{f/f}$* and *$Sh2b1^{\Delta SIM1}$* males (on chow diet, 18 weeks old) were injected with BDNF (60 ng/mouse, icv). VMH extracts were prepared 20 min later and immunoblotted with the indicated antibodies. Phosphorylation of ERK1/2 was normalized to total ERK1/2 levels. *$Sh2b1^{f/f}$*: n=3, *$Sh2b1^{\Delta SIM1}$*: n=3. **(C)** *$Sh2b1^{f/f}$* males (on normal chow diet) were bilaterally microinjected with AAV8-hSyn-fDIO-Cre or AAV-EF1a-GFP vector into the PVH and with retrograde AAV-EF1a-Flpo vector into the DRN. Ten weeks later, mice were stimulated with BDNF (40 ng/mouse, icv) for 20 min. PVH extracts were immunoblotted with antibodies to phospho-ERK1/2 and phospho-AKT. Phosphorylation of ERK1/2 and AKT were normalized to total ERK1/2 and AKT, respectively. AAV-EF1a-GFP: n=3, AAV-hSyn-fDIO-Cre: n=3. a.u.: arbitrary units. **(D)** *Sh2b1-Cre* males were bilaterally microinjected with AAV9-CAG-DIO-SH2B1 or AAV9-hSyn-DIO-mCherry vector into the PVH, fed a HFD for 10

weeks, and stimulated with BDNF (20 ng/mouse, icv) for 20 min. VMH extracts were immunoblotted with the indicated antibodies. Phosphorylation of AKT or ERK1/2 was normalized to total ERK1/2 or AKT levels, respectively. AAV-DIO-mCherry: n=3, AAV-DIO-SH2B1: n=3. **(E)** C57BL/6J males were bilaterally microinjected with AAV9-CAG-DIO-SH2B1 or AAV-CAG-DIO-GFP vector into the PVH and with retrograde AAV-hSyn-Cre vector into the DRN. One week later, the mice were fed HFD for 10 weeks. PVH extracts were immunoblotted with the indicated antibodies. Data are presented as mean ± SEM. *p<0.05, 2-tailed unpaired Student's *t* test.

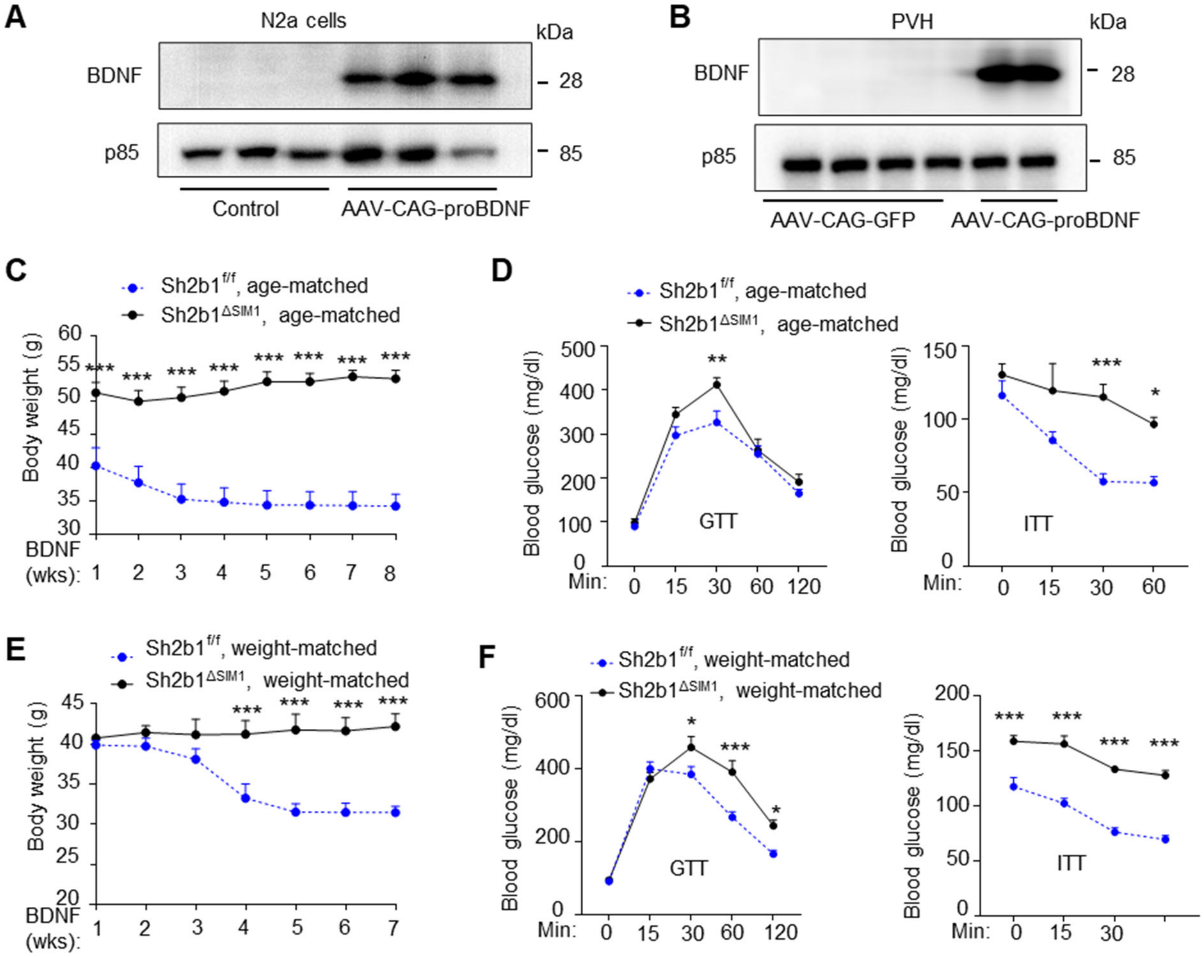

Figure S9. SH2B1 mediates BDNF's anti-obesity action in the PVH. (A) AAV9-CAG-proBDNF plasmids were transfected into N2a cells. Cell extracts were prepared 48 h after transfection and immunoblotted with antibodies to BDNF and p85 (PI 3-kinase regulatory subunit, loading control). **(B)** AAV9-CAG-proBDNF or AAV9-CAG-GFP vectors were microinjected into the PVH. PVH extracts were prepared 4 weeks after AAV transduction and immunoblotted with antibodies to BDNF and p85. **(C-D)** AAV9-CAG-proBDNF vectors were bilaterally injected into the PVH in $Sh2b1^{f/f}$ and $Sh2b1^{\Delta SIM1}$ males at 20 weeks of age (on normal chow diet). **(C)** Growth curves. $Sh2b1^{f/f}$: n=6, $Sh2b1^{\Delta SIM1}$: n=6. **(D)** GTT and ITT in 6 weeks after AAV transduction. $Sh2b1^{f/f}$: n=6, $Sh2b1^{\Delta SIM1}$: n=6. **(E-F)** $Sh2b1^{f/f}$ males were fed a HFD for 6 weeks. $Sh2b1^{\Delta SIM1}$ males were fed a normal chow diet. Body weight matched mice were bilaterally microinjected with vectors. **(E)** Growth curves. $Sh2b1^{f/f}$: n=9, $Sh2b1^{\Delta SIM1}$: n=6. **(F)** GTT and ITT in 6 weeks after AAV transduction. $Sh2b1^{f/f}$: n=7 for GTT and n=6

for ITT, *Sh2b1*$^{\Delta SIM1}$: n=6. Data are presented as mean ± SEM. *p<0.05, **p<0.01, ***p<0.001 two-way ANOVA.

ANTIBODY	SOURCE	Cat#	Blot	IHC
Actb	Abclonal	AC026	1:20000	
pAKT (pThr308)	Cell Signaling Technology	2965	1:1000	
pAKT (pSer473)	Cell Signaling Technology	4060	1:1000	
AKT	Cell Signaling Technology	2920	1:1000	
pERK1/2	Cell Signaling Technology	4370	1:1000	
ERK1/2	Santa Cruz	Sc-154	1:1000	
BDNF	ABclonal	A11028	1:1000	
SH2B1	Santa Cruz	Sc-136065		1:500
SH2B1	Home made		1:2000	
GFP	Cell Signaling Technology	2956		1:1000
c-Fos	Cell Signaling Technology	2250		1:1000
Tyrosine hydroxylase	Cell Signaling Technology	2792	1:1000	
p85	Home made		1:2000	

Table S1. Antibody list

Genes	Forward	Reverse
Pparγ	CCAGAGTCTGCTGATCTGCG	GCCACCTCTTTGCTCTGATC
36B4	AAGCGCGTCCTGGCATTGTCT	CCGCAGGGGCAGCAGTGGT
Prdm16	AGCAGCTGAGGAAGCATTT	GCGTGGAGAGGAGTGTCTTC
IL6	AGCCAGAGTCCTTCAGA	GGTCCTTAGCCACTCCT
Il1β	GCCTTGGGCCTCAAAGGAAAGAATC	GGAAGACACAGATTCCATGGTGAAG
Mcp1	ACTGAAGCCAGCTCTCTCTTCCTC	TTCCTTCTTGGGGTCAGCACAGAC
Fasn	TTGACGGCTCACACACCTAC	CGATCTTCCAGGCTCTTCAG
Acc1	CAGGGACTATGTCCTGAAGCA	GGAATCCATTGTGGAGAGGA
Tnfα	CATCTTCTCAAAATTCGAGTGACAA	TGGGAGTAGACAAGGTACAACCC
Pgc1a	TGGACGGAAGCAATTTTTCA	TTACCTGCGCAAGCTTCTCT
Ucp1	ATACTGGCAGATGACGTCCC	GTACATGGACATCGCACAGC
CD36	GGAGTGGTGATGTTTGTTGCT	GCACACACCACCATTTCTTCT
Srebp1c	AACGTCACTTCCAGCTAGAC	CCACTAAGGTGCCTACAGAGC
Chrebp	CTGGGGACCTAAACAGGAGC	GAAGCCACCCTATAGCTCCC
Dgat1	CGTGGTATCCTGAATTGGTG	GGCGCTTCTCAATCTGAAAT
Dgat2	ATCTTCTCTGTCACCTGGCT	ACCTTTCTTGGGCGTGTTCC

Table S2. Primer list
